# Surface Characterization and Antimicrobial Capability Evaluation of Medical-Grade Titanium Modified by Facile Immersion in the Solution of Novel Catechol-Terminated Compounds Having Cationic Quaternary Ammonium Functionality with Different Alkyl Chain Lengths

**DOI:** 10.3390/jfb17060271

**Published:** 2026-06-01

**Authors:** Zong-Hua Liu, Nai-Chia Fan, Chi-Hui Cheng, Jui-Che Lin

**Affiliations:** 1 Department of Chemical Engineering, National Cheng Kung University, Tainan 701, Taiwan; hansliu14@gmail.com; 2 Division of Nephrology, Department of Pediatrics, Chang Gung Memorial Hospital, Taoyuan 333, Taiwan; fannaichia@gmail.com (N.-C.F.); pedneph.cheng@msa.hinet.net (C.-H.C.); 3 Graduate Institute of Clinical Medical Sciences, Chang Gung University, Taoyuan 333, Taiwan; 4 Department of Pediatrics, College of Medicine, Chang Gung University, Taoyuan 333, Taiwan; 5 School of Dentistry, Institute of Oral Medicine, College of Medicine, National Cheng Kung University, Tainan 701, Taiwan

**Keywords:** cationic functionality, quaternary ammonium group, antibacterial, mussel-inspired, surface modification

## Abstract

Reducing hospital-acquired infections, especially those related to medical devices, is essential not only to improve patients’ well-being but also to reduce healthcare costs. Among various antibacterial approaches, creating bactericidal device surfaces has been advocated as it reduces the likelihood of antibiotic-resistant strains emerging when antibiotics are used. Functionalizing the device surface with cationic groups, such as quaternary ammonium terminal groups, has been considered an effective approach for killing microbes upon contact. Nonetheless, multiple steps, some of which may require harsh chemical reactions and toxic solvents, are generally required to attach the cationic quaternary ammonium functionalities to the surface. Inspired by the mussel’s capability to bind to various substrates, various novel biomimetic cationic catechol-terminated small molecules having the quaternary ammonium functionality with different alkyl chain lengths were synthesized for the first time. These compounds were used for surface modification of medical-grade titanium using simple immersion approaches: a single-layer procedure or a two-layer approach, in which the first layer was prepared by dopamine immersion, followed by a second immersion in the compound of interest. The surface characteristics and antimicrobial capability against the Gram-negative *E. coli* and Gram-positive *S. aureus* were assessed. The likely effects of the alkyl chain length and modification schemes on the surface properties and antibacterial activity are discussed and compared. The highest antimicrobial activity against *E. coli* was noted on the modified surfaces prepared by the two-layer approach with the cationic compound having the shortest alkyl chain, C1, at 2 mg/mL (DA_C1-2) and 8 mg/mL (DA_C1-8). The DA_C1-8 surface also exhibited the highest antimicrobial activity against *S. aureus*. These findings indicated that the antibacterial activity of titanium can be greatly improved by selecting the appropriate compound and a proper, facile immersion procedure.

## 1. Introduction

Nosocomial infection remains a critical issue in healthcare. Various attempts have been made to reduce its incidence rate, including strict enforcement of sterile medical practices and the use of sterilized medical equipment. Among these nosocomial infections, those associated with medical devices have been the focus of industry, academic research institutions, and clinical practitioners for decades. Despite advances in sterilization methods for medical devices and careful antiseptic clinical procedures, microbes, and even, sadly, antibiotic-resistant microbes, still have the chance to attach to device surfaces and subsequently proliferate, leading to infections of varying clinical severity. Henceforth, creating a surface that can reduce bacterial adhesion or even kill adhered microbes has been an ongoing challenge for device manufacturers and the academic research community [1,2,3,4,5,6,7].

Different methods have been investigated to reduce the number of viable bacteria. Nonetheless, fear of the emergence of antibiotic-resistant strains has restrained the widespread use of controlled antibiotic release from the medical device. On the other hand, killing the microbes once in contact with the surface to render the surface bactericidal has been considered as an effective alternative. Among various approaches to creating a bactericidal medical device surface, functionalizing the surface with cationic groups, such as quaternary ammonium, pyridinium, and phosphonium, has been considered the most effective technique. However, multistep chemical reactions and/or harsh reaction conditions were often required [2,8,9,10]. Among these cationic functional groups, quaternary ammonium groups were commonly employed due to their environmental stability, low toxicity, and permanent contact-active properties [2,3,11,12].

Different methods have been proposed to incorporate cationic surface functionalities. The layer-by-layer method has been utilized to render the surface positively charged. Although the modification procedure is easy to handle, the use of coupling agents to keep the interlayers covalently bound was necessary to reduce the likelihood of dissociation between the ionic interlayers due to competitive ionic interactions from small ions in physiological conditions [13]. Grafting-to, grafting-from, and grafting-through approaches have also been used to functionalize the surface with cationic groups [14]. All three approaches require a surface pre-activation reaction to generate surface free radical sites or functional groups for subsequent reaction with the compound bearing cationic functionalities of interest. But multiple complicated reaction steps, surface grafting density control, and grafting chain length multiplicity, to name a few [15,16,17,18,19], have made the three approaches difficult to scale up for commercial production.

On the other hand, due to its capability to reduce the use of harsh chemical reactions and toxic/organic solvents, the mussel-inspired surface modification technique has been a research focus because of the mussel’s capability to bind to various organic and inorganic substances in the ocean or along the coastline [20,21,22,23,24,25]. Different reaction schemes, such as π-π stacking, cation-π interactions, hydrogen bonding, cohesive bonding, hydrophobic–hydrophobic interactions, ionic interactions, and covalent bonding, have been attributed to the formation of mussel-like catechol- or dopamine-based deposits on organic plastics and inorganic metallic substrates, depending on the depositing compounds and the substrate of selection [26,27,28,29,30,31,32].

Inspired by the mussel’s adhesive capability, our group has recently synthesized a novel biomimetic catechol-terminated “polymer” with cationic quaternary ammonium functionalities as an antibacterial coating for medical-grade titanium and polypropylene using simple immersion treatment schemes [3]. In addition, previous studies have reported that the alkyl chain length of quaternary ammonium compounds can affect their antibacterial activity [2,33,34,35,36,37]. Henceforth, to further broaden these findings, various novel mussel-inspired catechol-terminated “small” molecules having the cationic quaternary ammonium functionality with different alkyl chain lengths were synthesized. These compounds were used to modify medical-grade titanium substrates via different solution-immersion methods.

Previous studies have shown that dopamine can assist or mediate the subsequent deposition/coating process. Dopamine can be added to the coating solution as a mixture to improve the coating process [3,4,38]. On the other hand, dopamine can be deposited as a first layer to assist the subsequent modification process due to its universal adhesive properties and reactive functionalities [3,4,38,39,40]. In view of these findings, besides the direct coating of the piranha acid-oxidized/cleaned titanium substrate by different cationic quaternary ammonium catechol-terminated compounds (single-layer approach), a layer of polydopamine was pre-deposited on the substrate before coating with the cationic catechol-terminated compounds of interest (two-layer approach). The surface characteristics and antibacterial capability against gram-negative *E. coli* and gram-positive *S. aureus* of these modified titanium substrates were evaluated and optimized. Moreover, the likely effects of the immersion scheme (single-layer vs. two-layer) and alkyl chain length (C8, C4, and C1) on the surface properties and antimicrobial activity of the modified titanium substrates were compared and discussed.

## 2. Materials and Methods

### 2.1. Materials

Chemicals required to synthesize novel catechol compounds with quaternary ammonium groups at different alkyl chain lengths, as well as for the subsequent surface modification of the titanium substrate, were procured from different vendors at the highest available purity. Dopamine hydrochloride (DA), tetrahydrofuran, tris(hydroxymethyl)aminomethane (Trizma base), and hydrogen peroxide were acquired from Sigma-Aldrich (St. Louis, MO, USA). Methyl trifluoroacetate (MTFA), 2,2-dimethoxypropane (DMP), p-toluenesulfonic acid (PTSA), potassium carbonate, and 4-(dimethylamino)pyridine (DMAP) were from Alfa Aesar (Ward Hill, MA, USA). Triethylamine (TEA), hydrochloric acid, and sulfuric acid were from J.T. Baker (Radnor, PA, USA). Octanoic acid, chlorotrimethylsilane (TMSCl), lithium aluminum hydride 2.4 M solution in THF (LiAlH_4_), and trifluoroacetic acid (TFA) were from Thermo Scientific (Waltham, MA, USA). Calcium chloride and iodomethane were from SHOWA (Tokyo, Japan). 1-(3-Dimethylaminopropyl)-3-ethylcarbodiimide Hydrochloride (EDC) was from Tokyo Chemical Industry (Tokyo, Japan). Sodium bicarbonate and sodium sulfate were purchased from Fluka (Charlotte, NC, USA). Benzene, methanol, and ethyl acetate were from MACRON (Radnor, PA, USA). Dichloromethane, chloroform, and hexane were from DUKSAN (Ansan, Republic of Korea). N-butyric acid and formic acid were from Riedel-de Haën (Seelze, Germany). Toluene was from TEDIA (Fairfield, OH, USA).

Anion exchange resin (Amberlite^TM^ IRA-402, Cl-form) was from Thermo Scientific (Waltham, MA, USA). The silica gel (70–230 mesh, pH 7.0) was obtained from SiliCycle Inc. (Quebec, QC, Canada). The titanium substrate (grade I c.p. Ti, thickness 0.2 cm) was acquired from AcrUshin Co. (Taipei, Taiwan).

For the antibacterial assay, soya peptone and tryptone type 1 were obtained from HIMEDIA (Maharashtra, India). Agar was purchased from Becton, Dickinson and Company (Franklin Lakes, NJ, USA). Sodium chloride, sodium hydroxide, and ethanol were acquired from Sigma-Aldrich (St. Louis, MO, USA), Honeywell Research Chemicals (Charlotte, NC, USA), and J.T. Baker (Radnor, PA, USA), respectively, at their highest grade/purity available. The microbial strains, namely *E. coli* (ATCC 23501) and *S. aureus* (ATCC 21351), were acquired from the Food Industry Research and Development Institute in Hsinchu, Taiwan.

### 2.2. Synthesis

**Figure 1:**
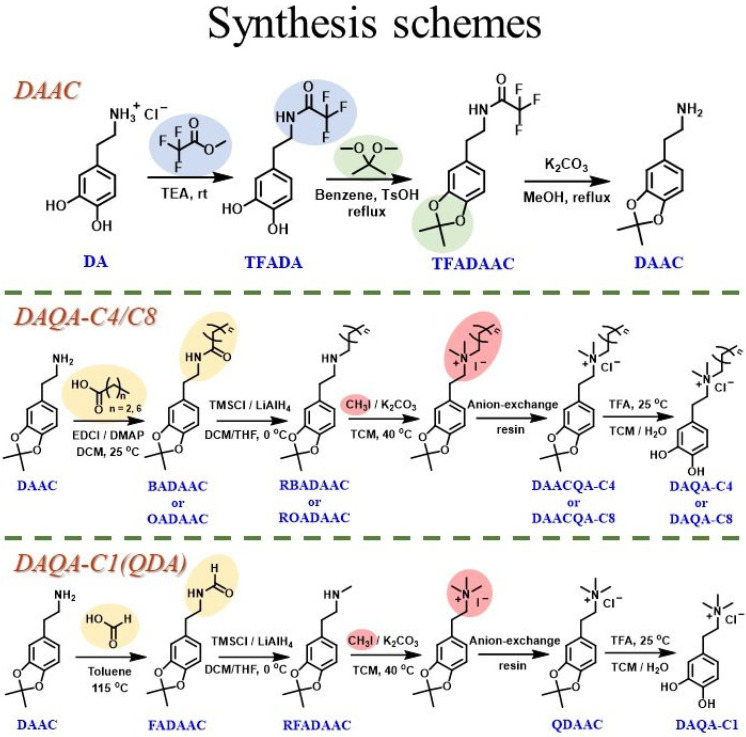
The synthesis schemes for quaternary ammonium compounds with different alkyl chain lengths, namely DAQA-C8, DAQA-C4, and DAQA-C1 (QDA).

The synthesis schemes for catechol-terminated compounds with quaternary ammonium functionality at different alkyl chain lengths, namely DAQA-C8, DAQA-C4, and DAQA-C1 (QDA), are shown in Figure 1.

#### 2.2.1. Synthesis of DAAC

Dopamine-HCl (105 mmol), 20 g, was dissolved in 350 mL of methanol and added to a round-bottom flask under argon. Then, 23 mL of methyl trifluoroacetate (MTFA, 229 mmol) and 64 mL of triethylamine (TEA, 459 mmol) were added to the flask. The reaction was carried out at room temperature for 24 h. After removal of the solvent, the mixture was adjusted to pH 3–4 with 1 N HCl (aq), then extracted with ethyl acetate several times, washed with brine, and dried over Na_2_SO_4_, and then the solvent was removed under vacuum. The brown solid **TFADA** with a yield of 99% was obtained.

To protect the catechol group, the following step was carried out. **TFADA** (28 mmol), 7 g, was dissolved in 400 mL benzene and added to a three-neck round-bottom flask, and 15 mL 2,2-dimethoxypropane (122 mmol) was added to the flask. The reaction system was using a Soxhlet extractor to remove the byproducts. The Soxhlet thimble was filled with 30 g of anhydrous CaCl_2_ to absorb water and methanol. After the first reflux, 0.2617 g *p*-Toluenesulfonic acid (TsOH, 1.38 mmol) was added to the flask. The reaction was carried out overnight and could be monitored by the FeCl_3_ test on a TLC plate. The solvent was removed and passed through silica gel chromatography (*V*_ethyl acetate_:*V*_hexane_ = 1:10 + 3% TEA). The final product was recrystallized from hexane and dried under vacuum. The white crystal **TFADAAC** with 80% yield was obtained.

**TFADAAC** (20.7 mmol), 6 g, was dissolved in 180 mL of methanol and added to a round-bottom flask. K_2_CO_3_ (62.1 mmol), 8.58 g, and 5 mL deionized (DI) water were added to the flask. It was heated up to reflux temperature, and the reaction was monitored by TLC plate. After filtration of the mixture and removal of the solvent, the product was dissolved in DI water, extracted with chloroform several times, washed with DI water, dried over Na_2_SO_4_, and dried in a vacuum oven overnight. A brownish liquid of **DAAC** with 94% yield was obtained.

#### 2.2.2. Synthesis of DAQA-C8

1-(3-Dimethylaminopropyl)-3-ethylcarbodiimide Hydrochloride (EDC, 40.8 mmol), 7.83 g, and 0.3 g 4-(dimethylamino)pyridine (DMAP, 2.45 mmol) were dissolved in 80 mL dichloromethane (DCM) and added to a three-neck round-bottom flask. **Octanoic acid** (24.5 mmol), 3.54 g, was then added, and the reaction was allowed to proceed at room temperature for 5 min. **DAAC** (26 mmol), 5 g, was dissolved in 45 mL of dichloromethane and then added to a cylinder isobaric separatory funnel. DAAC was slowly added to the bottom flask, and the reaction was continued for 24 h at room temperature. After monitoring the reaction by TLC plate, the product mixture was extracted with 1 M HCl, DI water, saturated sodium bicarbonate, and saturated brine, in sequence. The solvent was removed, and the organic phase was further purified by silica gel chromatography (*V*_ethyl acetate_:*Vhexane* = 1:15). The eluent was removed, and the product was dried under vacuum overnight. Off-white solid of **OADAAC,** 6.3 g, was obtained in 80.4% yield.

**OADAAC** (26.6 mmol) solid, 8.5 g, was added to a three-neck round-bottom flask. After setting up two isobaric separatory funnels, 130 mL of dichloromethane and 50 mL of THF were drained into the first separatory funnel and added to the flask. Chlorotrimethylsilane (TMSCl, 157 mmol), 20 mL, was drained to the second separatory funnel and then added to the flask at 0 °C. After reacting for 1 h, 35 mL of Lithium Aluminum hydride in 2.4 M THF solution (LiAlH_4_, 84 mmol) was drained to the first separatory funnel and then slowly added to the flask at 0 °C. After 24 h of reaction, the product solution mixture was extracted with saturated sodium bicarbonate and saturated brine aqueous solution in sequence; then, the organic phase mixture was purified by passing through silica gel chromatography (*V*_ethyl acetate_:*V*_hexane_ = 1:10). After that, the eluent was removed and dried in a vacuum overnight, and 6.3 g light yellow liquid of **ROADAAC** was obtained in 61.5% yield.

**ROADAAC** (15.4 mmol), 4.7 g, was added to a three-neck round-bottom flask containing 60 mL chloroform (trichloromethane, TCM) after 7.3 g of potassium carbonate (52.6 mmol) was added to the flask. Iodomethane (107.6 mmol), 6.7 mL, was then slowly added to the reaction flask, and the reaction was allowed to proceed at 50 °C for 2 days. After the reaction, the organic phase was extracted with DI water 4–5 times, the organic solvent was removed, and the product was dried in a vacuum at 50 °C overnight. The iodine was then exchanged with Cl using the anionic resin Amberlite IRA-402 (Cl-form) in methanol. Off-white **DAACQA-C8** solid, 6.8 g, was obtained in 95.7% yield.

**DAACQA-C8** (12.9 mmol), 4.8 g, was dissolved in 100 mL chloroform and added to a three-neck round-bottom flask, followed by the addition of 8 mL DI water and 28 mL trifluoroacetic acid (TFA, 366 mmol); then, the reaction was heated at 30 °C for 3 h. After the reaction, the solvent was removed, and the product was dried under vacuum at 50 °C overnight. A brown liquid of **DAQA-C8** with a 99.5% yield was obtained.

#### 2.2.3. Synthesis of DAQA-C4

EDC (33.9 mmol), 6.5 g, and 0.3 g DMAP (2.45 mmol) were dissolved in 90 mL of dichloromethane and added to a three-neck round-bottom flask. Butyric acid (22.7 mmol), 2 g, was then added, and the reaction was stirred at room temperature for 5 min. **DAAC** (30.5 mmol), 5.89 g, was dissolved in 45 mL of dichloromethane and then added to a cylinder isobaric separatory funnel. The DAAC was slowly added to the bottom flask. The reaction was continued for 24 h at room temperature. After monitoring the reaction by TLC plate, the product mixture was extracted with 1 M HCl, DI water, saturated sodium bicarbonate, and saturated brine, in sequence. The organic phase mixture was further purified by silica gel chromatography (*V*_ethyl acetate_:*V*_hexane_ = 1:10). The solvent was removed from the eluent, and the product was dried under vacuum overnight. Off-white solid of **BADAAC**, 4.1 g, was obtained in 68.6% yield.

**BADAAC** (28.5 mmol) solid, 7.5 g, was added to a three-neck round-bottom flask. After setting up two isobaric separatory funnels, 150 mL of dichloromethane and 50 mL of THF were drained to the first separatory funnel and added to the flask, and 22.5 mL of TMSCl (177 mmol) was drained to the second separatory funnel and then added to the flask at 0 °C. After reacting for 1 h, 75 mL of Lithium Aluminum hydride in 2.4 M THF solution (LiAlH_4_, 180 mmol) was drained to the first separatory funnel and then slowly added to the flask at 0 °C. After 24 h of reaction, the product solution mixture was extracted with saturated sodium bicarbonate and saturated brine aqueous solution in sequence; then, the organic phase mixture was purified by passing through silica gel chromatography (*V*_ethyl acetate_:*V*_hexane_ = 1:8). After that, the eluent was removed and dried in a vacuum overnight. Light yellow liquid of **RBADAAC**, 5 g, was obtained in 70% yield.

**RBADAAC** (14.8 mmol), 3.7 g, was added to a three-neck round-bottom flask containing 45 mL chloroform (TCM), after which 6.2 g potassium carbonate (44.8 mmol) was added. Iodomethane (89.9 mmol), 5.6 mL, was then slowly added to the reaction flask, and the reaction was allowed to proceed at 50 °C for 2 days. After the reaction, the organic phase was extracted with DI water 4–5 times, the organic solvent was removed, and the product was dried in a vacuum at 50 °C overnight. The iodine was then exchanged with Cl using the anionic resin Amberlite IRA-402 (Cl-form) in methanol. Off-white solid of **DAACQA-C4,** 2.5 g, was obtained in 52.7% yield.

**DAACQA-C4** (8 mmol), 2.5 g, was dissolved in 65 mL chloroform and added to a three-neck round-bottom flask, followed by the addition of 6 mL DI water and 17 mL trifluoroacetic acid (TFA, 366 mmol). The reaction was heated at 30 °C for 3 h. After the reaction, the solvent was removed, and the product was dried under vacuum at 50 °C overnight. A brown liquid of **DAQA-C4** with a yield of 95.1% was obtained.

#### 2.2.4. Synthesis of DAQA-C1 (QDA)

After setting up the Dean–Stark apparatus, 8.3 g **DAAC** (43 mmol) and 2.97 g **Formic acid** (64.5 mmol) were added to a round-bottom flask containing 200 mL of toluene, and the mixture was heated to 110 °C for 24 h. After a 24 h reaction, the organic solvent was removed by rotavapor, and the raw product was redissolved in dichloromethane, then extracted with 1 M HCl, DI water, saturated sodium bicarbonate, and saturated brine. Then, the organic-phase mixture was purified by silica gel chromatography (*V*_ethyl acetate_:*V*_hexane_ = 1:6). After purification, the eluent was removed, and the mixture was dried under vacuum overnight. Orange liquid of **FADAAC**, 8.1 g, was obtained in 85.3% yield.

**FADAAC** (22.6 mmol) liquid, 5 g, was added to a three-neck round-bottom flask. After 130 mL of dichloromethane and 45 mL of THF was drained to the first separatory funnel and added to the flask, 20 mL of TMSCl (157 mmol) was drained to the second separatory funnel, then added to the flask at 0 °C. After reaction for 1 h, 65 mL of Lithium Aluminum hydride in 2.4 M THF solution (156 mmol) was drained in the first separatory funnel and slowly added to the flask at 0 °C. After 24 h of reaction, the product solution mixture was extracted with saturated sodium bicarbonate and saturated brine aqueous solution in sequence; then, the organic phase mixture was purified by passing through silica gel chromatography (*V*_ethyl acetate_:*V*_hexane_ = 1:4). After purification, the eluent was removed and dried in a vacuum overnight, and 3.4 g yellow liquid of **RFADAAC** was obtained in 72.6% yield.

**RFADAAC** (18.3 mmol), 3.8 g, was added to a three-neck round-bottom flask containing 90 mL of chloroform. After 2 g of potassium carbonate (14.5 mmol) was added to the flask, 5.6 mL of iodomethane (89.9 mmol) was slowly added to the reaction flask, which was heated at 50 °C for 2 days. After the reaction, the organic phase was extracted with DI water 4–5 times, the organic solvent was removed, and the product was dried in a vacuum overnight. The iodine was then exchanged with Cl using the anionic resin Amberlite IRA-402 (Cl-form) in methanol. Milk tea color solid of **QDAAC** (**DAACQA-C1**), 4.5 g, was obtained in 90.3% yield.

**QDAAC (DAACQA-C1)** (16.6 mmol), 4.5 g, was dissolved in 100 mL chloroform and added to a three-neck round-bottom flask, followed by adding 12 mL DI water and 35.8 mL trifluoroacetic acid (467 mmol), and reacted at 30 °C for 3 h. After the reaction, the solvent was removed, and the product was dried under vacuum at 50 °C overnight, yielding a brick-red solid **DAQA-C1** with a 98.7% yield.

### 2.3. Surface Modification of Titanium (Ti) Substrate

The titanium substrate was first cut into 1 × 1 cm^2^ sheets and cleaned with neutral detergent, DI water, ethanol, and acetone in sequence under ultrasonication for 15 min each, then blow-dried with nitrogen gas. The cleaned titanium was further immersed in Piranha solution for 1 h and then cleaned ultrasonically in DI for 15 min.

The Piranha-cleaned/oxidized titanium sheet was then immersed vertically in the coating solution by hanging it downward, without reaching the bottom of the solution glass beaker. The coating solutions were prepared at specific concentrations of quaternary compounds with different chain lengths in a 50 mM ethanol–tris buffer solution. The beaker was placed on a rotary shaker, and the coating was performed at 30 °C and 75 rpm for 24 h in the dark to avoid likely UV effects on the titanium surface and the activation/reaction of the catechol end of the coating compound. After that, the samples were ultrasonically cleaned with deionized water for 10 min, blown-dried with nitrogen, and then vacuum-dried for further analyses.

**Figure 2:**
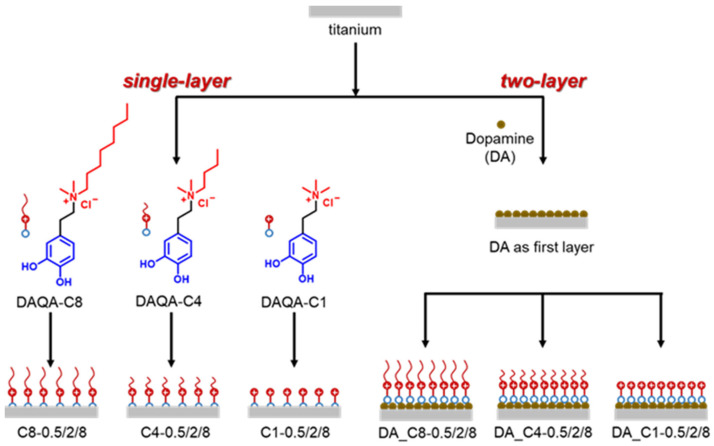
Single-layer and two-layer surface modification schemes.

Besides the direct coating with the single compound of interest (single-layer approach), the Piranha-cleaned/oxidized titanium substrate was first coated with 2 mg/mL dopamine for 4 h as the first layer, followed by coating with the compounds of interest (two-layer approach; Figure 2 and Table 1).

### 2.4. Characterization

The purity and chemical configurations of DAQA-C8, DAQA-C4, DAQA-C1, and the intermediates in the synthesis scheme were examined by nuclear magnetic resonance (NMR) spectroscopy (Bruker AV-500, Fällanden, Switzerland).

The sessile drop method by water contact angle meter (WCA) (Model 100SB, Sindatek Inc., Taipei, Taiwan) was utilized to determine the surface hydrophilicity of different titanium substrates after vacuum drying. The chemical bonding and elemental composition of different modified Ti substrates were characterized by X-ray photoelectron spectroscopy (XPS) (PHI Quantera II, ULAVAC-PHI, Chigasaki, Japan) using AlKα as the X-ray source (1486.6 eV), and the detector was set at a take-off angle of 45°, pass energy 55 eV, and step size 0.1 eV.

### 2.5. Antibacterial Test

Gram-negative *E. coli* (ATCC 23501) and Gram-positive *S. aureus* (ATCC 21351) were selected for the antibacterial assays due to their differences in cell wall structures. In addition, these two bacteria were also among the most cultured microbes from the medical-device-associated infection. Different modified samples were first sterilized with 75% ethanol and vacuum-dried. All samples were further sterilized by UV exposure before the antibacterial assay. The sample was then immersed in a tube containing 1 mL of bacterial suspension (2 × 10^6^ CFU/mL) and incubated at 37 °C under shaking at 150 rpm for 6 h in the dark to avoid the likely bactericidal effect of UV light [41].

After 6 h of bacterial incubation, the sample was immersed in 5 mL of 0.9% sterile PBS solution in an ultrasonicator at 200 W and 40 kHz for 10 min to remove surface-adhered bacteria. The detached bacteria solution was appropriately diluted and spread on agar plates to count viable colonies and assess antibacterial activity. The antibacterial activity was determined as the percentage reduction in bacterial growth compared to the non-modified control, bare Ti. Three specimens per category were tested.

## 3. Results and Discussion

### 3.1. DAQA-C8, DAQA-C4, and DAQA-C1

The ^1^H-NMR spectra of DAQA-C8, DAQA-C4, and DAQA-C1 are shown in Figure 3a, Figure 3b and Figure 3c, respectively. The ^1^H-NMR spectra of the intermediates shown in the synthesis scheme (Figure 1) are shown in Figures S1–S12. The NMR spectra indicated that all these compounds were well prepared and of reasonably high purity.

### 3.2. Surface Characterization

#### 3.2.1. Surface Wettability by Contact Angle Measurement

The static water contact angle, determined by the sessile drop method, was initially used to assess the completeness of different surface modification modalities on the titanium substrate. Due to the relatively hydrophobic chemical configurations of DAQA-C8 and DAQA-C4, a 1:1 (*v*/*v*) mixture of ethanol and deionized water was used as the solvent for dissolving the compound for coating.

**Figure 4:**
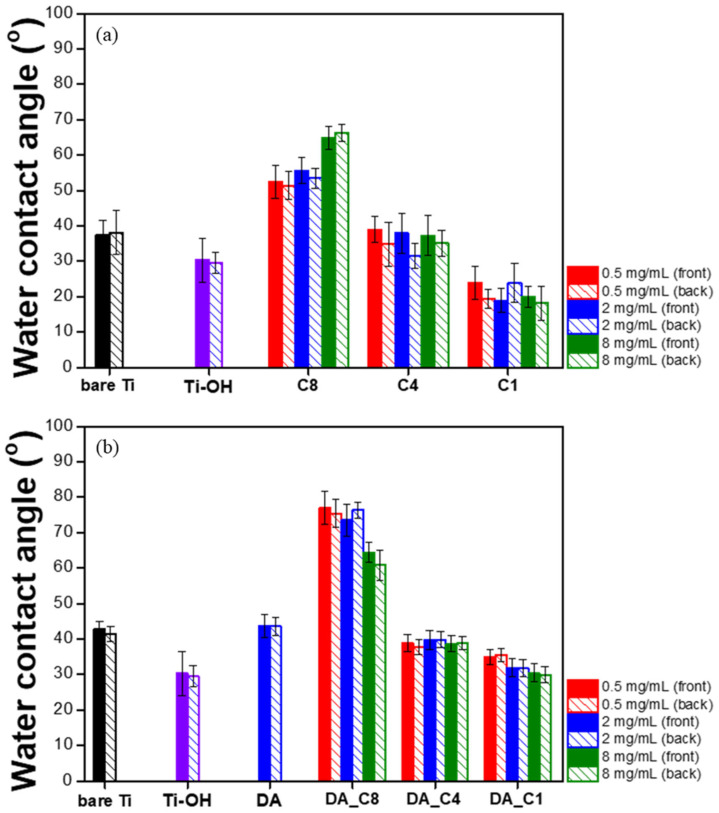
The water contact angle of titanium substrates modified by (**a**) single-layer approach, (**b**) two-layer approach. For the bare Ti, Ti-OH, and DA samples, the value for the front side was shown as a filled bar while the value for the back side was presented as the hatched bar.

Figure 4 shows the contact angles of titanium substrates modified by various schemes after vacuum drying. There is no significant difference in the water contact angles between the front and back surfaces of all samples, demonstrating that the double-sided coating design strategy is stable and feasible.

The results show that the unmodified titanium surface had a water contact angle of around 40° after drying (bare Ti). The higher contact angle value noted in the cleaned titanium substrate (bare Ti) could be due to the surface-adsorbed adventitious hydrocarbons (see XPS section) or the surface titanoxane (Ti-O-Ti) formed after being exposed to an oxygen-containing environment or irradiated with visible light [4,42]. After washing with a piranha solution to remove surface-adsorbed organic contaminants and increase the number of OH groups on the titanium surface (Ti-OH), the water contact angle decreased to approximately 30°.

Quaternary ammonium molecules were subsequently coated on the piranha acid-oxidized titanium surface (single-layer coating). The water contact angle values of various modified surfaces by the single-layer approach would decrease with the alkyl chain length of these three cationic catechol-terminated compounds (i.e., C8 > C4 > C1, Figure 4a). Furthermore, the contact angle variations with the concentration used for single-layer surface modification were observed only for those modified with DAQA-C8, specifically C8-8 > C8-2 ≈ C8-0.5. This may be attributed to the pronounced hydrophobic effects caused by the C8 alkyl chain in the DAQA-C8 than the C4 chain in the DAQA-C4 and C1 in the DAQA-C1on the modified surface. Furthermore, the higher water contact angles of the C8 and C4 single-layer modified surfaces than the Ti-OH indicated successful surface modification by these two cationic catechol-terminated compounds, DAQA-C8 and DAQA-C4. As for the surfaces modified with C1 (DAQA-C1), further XPS analysis (3.2.2 XPS analysis) would be needed to confirm the success of the surface modification, since the C1-modified surfaces were more hydrophilic than the Ti-OH substrate.

For the two-layer approach, the surface contact angle increased after the piranha-modified surface (Ti-OH) was immersed in the dopamine solution for 4 h (DA; Figure 4b). Subsequent immersion in different cationic quaternary ammonium compounds has led to distinct trends in variations of hydrophobicity. For these surfaces prepared by the two-layer approach, the surface hydrophobicity increased with the alkyl chain length (i.e., DA_C8 > DA_C4 > DA_C1), as in the single-layer approach. Nevertheless, only the DA_C8 surfaces showed contact angles higher than those of the substrate coated with dopamine (DA), while the other two, DA_C4 and DA_C1, surfaces exhibited lower contact angles. This trend differed significantly from that observed in the single-layer approach, in which only the samples modified with DAQA-C1 exhibited a contact angle lower than that of the substrate, Ti-OH. Moreover, concentration-dependent variations in contact angle were observed in the DA_C8 and DA-C1 series, but not in the DA_C4 series. For the DA_C8 and DA_C1 series, the one modified by the lower concentration of DAQA_C8 and DAQA_C1 would exhibit a higher contact angle, DA_C8-8 < DA_C8-2 ≈ DA_C8-0.5, and DA_C1-8 ≈ DA_C1-2 < DA_C1-0.5. This concentration-dependent variation differs from that observed in the single-layer approach.

This distinct concentration-dependent variation could be attributed to the differences in the substrate used for the single-layer, Ti-OH, and two-layer approaches, DA. The Ti-OH presented a more hydrophilic surface than the dopamine-coated substrate (DA), by which the catechol groups and cationic terminal ends associated with the cationic quaternary ammonium coating compounds would be more favorable to interact with the -OH or titanol groups on the Ti-OH surface while leaving the hydrophobic ends of the coating compounds facing outwards, especially the one with the longest alkyl chain length, C8. Henceforth, increasing the concentration of the DAQA-C8 compound in the coating solution would result in a higher contact angle in the single-layer approach. On the other hand, for the two-layer approach, the hydrophobic alkyl chains would be more favorable for interacting with the hydrophobic dopamine-coated substrate (DA), while the more hydrophilic ends would reside in the outer layer. This would result in a lower contact angle if higher concentrations of the coating solutions DAQA-C8 and DAQA-C1 were used. Nevertheless, the balanced interactions between the C4 alkyl chains of DAQA-C4 and the dopamine first layer would yield similar contact angles for the two-layer-modified DA_C4 surfaces prepared with different concentrations.

#### 3.2.2. XPS Analysis

The surface atomic percentages of various modified titanium substrates modified by the single-layer and two-layer approaches are shown in Table 2. The cationic quaternary C_4_N^+^% was determined by the N1s atomic percentage times the C_4_N^+^ area percentage value derived from the N1s curve fitting. The N1s peak was deconvoluted to the Ti-N (397 eV), C-N (399.7 eV), and C_4_N^+^ (402.4 eV) for all samples [43,44], and the full width at half maximum (FWHM) value of each deconvoluted peak was less than 1.8 eV.

The C1s, N1s, and O1s peaks were observed alongside the Ti2p peak on the non-piranha solution-oxidized titanium (bare Ti). This is likely due to adsorbed adventitious hydrocarbon, surface titanoxane (Ti-O-Ti) formed after exposure to an oxygen-containing environment or irradiation with visible light, or organic matter attached to the titanium surface during titanium sheet processing [45]. After the piranha solution oxidation, lower C1s and N1s atomic percentages and higher O1s atomic percentage were noted, likely due to the removal of adsorbed hydrocarbon as well as the formation of OH functional groups on the Ti-OH substrate surface. Furthermore, after Piranha solution cleaning/oxidation, the reduction of N1s was much greater than that of C1s, and the N1s atomic percentage decreased to less than 1%. This implies that the C1s on the Ti-OH may be attributed to the hard-to-remove hydrocarbons containing mainly the C atoms, which were added/adhered during titanium sheet processing. In contrast, the hydrocarbon-containing N1s signals noted on the Ti substrate were highly likely to be removed after the Piranha immersion.

##### Surfaces Modified by the Single-Layer Approach

For the surfaces modified by the single-layer approach, lower Ti2p and higher C1S atomic percentage values were observed compared to the substrate, Ti-OH. Furthermore, for those modified by the compound with a longer alkyl chain, DAQA-C8, the Ti2p signals decreased significantly with increasing coating concentration, suggesting that coating thickness increased with concentration, especially for those modified by C8-8. On the other hand, no significant variation in Ti2p atomic concentration was noted on the surfaces modified by DAQA-C4 and DAQA-C1.

For the surface cationic quaternary ammonium percentage (C_4_N^+^), the C8-8 modified surface presented the highest surface cationic quaternary ammonium percentage (C_4_N^+^), as well as the significantly highest C_4_N^+^/N1s ratio (0.71 vs. 0.2–0.4) among the surfaces modified by the single-layer approach, suggesting this C8-8 surface is well packed/grafted with DAQA-C8. As for the surfaces modified by the DAQA-C4 at different concentrations, these surfaces presented a lower C_4_N^+^ percentage than the DAQA-C8 and DAQA-C1, which may result from the differences in the competitive interactions between (1) the solutes and solvent (50 mM ethanol–tris buffer solution) and (2) the solutes and Ti-OH substrate. DAQA-C8 presented a longer alkyl chain, which could lead to less thermodynamically favorable interactions with polar molecules, such as water and ethanol, in solution. As a result, it is easier to graft or deposit onto the Ti-OH substrate. On the other hand, DAQA-C1, the one with the shortest alkyl chain, would likely act as the dopamine and is easy to react with the Ti-OH substrate. Nevertheless, the interactions between the DAQA-C1 and solvent molecules (ethanol and water) would also be thermodynamically favorable due to the short alkyl chain, C1. As compared to the DAQA-C8 and DAQA-C1, the interactions of DAQA-C4 with the Ti-OH substrate may be less than those of these two counterparts, while the interactions of DAQA-C4 with ethanol and water solvents may be more favorable than those of DAQA-C8. Henceforth, the competition between the deposition/grafting process and solvation would determine the amount of solute deposited/grafted on the Ti-OH substrate.

##### Surfaces Modified by the Two-Layer Approach

For the two-layer approach, the first layer, prepared by depositing dopamine on the Ti-OH substrate (i.e., DA), was fairly thick, exceeding a few hundred angstroms or even a few microns, to block the Ti2p signals from reaching the XPS detector. Meanwhile, the C1s and N1s atomic percentages increased while the O1s atomic percentage decreased after the deposition of polydopamine.

Instead of a thin surface deposit observed with the single-layer approach, the deposit prepared by the two-layer approach was found to be fairly thick, as indicated by the disappearance or very low atomic percentage of Ti2p signals from the substrate.

After the second layer deposition, the surfaces formed by DAQA-C8 deposition, as expected, showed the highest C1s values than those formed by DAQA-C4 and DAQA-C1 due to the longer alkyl chain length. Nevertheless, only those by the DAQA-C8 presented a higher C1s value than the DA, the surface with the first layer. This suggests that the hydrophobic–hydrophobic interactions/attractions between the first-layer deposited polydopamine and the alkyl chain of DAQA-Cx (x = 8, 4, and 1) would govern the second-layer deposition. The DAQA-C8 has the longest alkyl chain and, as a result, would be easier to deposit onto the polydopamine first layer.

In contrast to the surfaces prepared by the single-layer approach, the N1s atomic percentage of the surfaces prepared by the two-layer approach decreased with the increase in the concentration of DAQA-Cx. Nevertheless, the cationic quaternary ammonium percentage (C_4_N^+^) and C_4_N^+^/N1s ratio increased with the concentration of DAQA-Cx, as did those prepared by the single-layer approach. This indicates that these cationic C_4_N^+^ functionalities would reside in the top few hundred angstroms layer due to the thermodynamically unfavorable interactions between these cationic functional groups and the hydrophobic polydopamine first layer. This would partially explain the contact angle findings on the surfaces modified by the two-layer approach using DAQA-C8 and DAQA-C1, in which the lowest contact angle value was observed at the highest concentration, 8 mg/m. On the other hand, the findings regarding the increase in C4N^+^ with increasing DAQA-Cx concentration reflect the overall amount of DAQA-Cx deposited on the Ti-OH substrate, given its low thickness. Similar to the discussion shown above for the single-layer modification, these two-layer deposition findings would be likely attributed to the competitive interactions between (1) the solutes, DAQA-Cx, and solvent (50 mM ethanol–tris buffer solution), and (2) the solutes and the hydrophobic first-deposited layer, DA.

##### Curve Fitting for the C1s of the Surfaces Modified by the Single-Layer and Double-Layer Approach

To further delineate the surface carbon chemical binding state, the C1s peak was deconvoluted into the following four types of bonding [46,47,48]: (1) C-C/C-H/C=C (285 eV); (2) C-O/C-N (286.6 eV); (3) C=O (288.6 eV); and (4) Ti-C (282 eV). The full width at half maximum (FWHM) of each deconvoluted peak is below 1.8 eV (Figure S13). The percentage of the deconvoluted peak area is shown in Table 3.

The Ti-C noted on bare Ti was speculated to be due to hydrocarbon contaminants during titanium sheet processing [45]. These pollutants cannot be removed by alcohol, acetone, or neutral detergents, so piranha acid is used to clean the surface pollutants. After cleaning the titanium surface with piranha acid (i.e., Ti-OH), in addition to the disappearance of Ti-C bonds, other carbon bonds remained on the surface. It is speculated that this may be because when the piranha acid dissolves the organic pollutants, hydrogen peroxide cannot convert the hydrocarbon pollutants into carbon dioxide in time, so the dissolved organic matter reacts with the titanium etched on the surface. In addition, the increase in the C-O percentage may be due to oxidation of the organic matter that was not dissolved/removed from the surface by piranha acid, so the surface now has new C-OH functional groups. This finding is substantiated by the XPS elemental composition shown in Table 2 for Ti-OH, in which the C1s percentage was decreased while the O1s was increased following the piranha acid cleaning. This indicated that piranha acid can significantly reduce the amount of organic pollutants on the titanium surface and increase the number of OH functional groups.

Subsequent single-layer coatings of quaternary ammonium molecules with different alkyl chains, DAQA-Cx, onto the Ti-OH surface (Table 3) revealed that all modified samples exhibited more C-O/C-N bonds than Ti-OH. Furthermore, in the DAQA-C8 and DAQA-C4 groups, the C-O bond ratio increased with coating concentration, indicating greater molecular deposition/grafting with higher concentrations of cationic solutes in solution. Nevertheless, due to the thin deposit layer, as indicated by the notice of Ti2p signals from the substrate in Table 2, it is difficult to precisely determine the schematic mechanism of single-layer deposition based on the C1S curve-fitting results (Table 3). Further, the reappearance of Ti-C on surfaces modified by the single-layer approach with DAQA-C4 and DAQA-C1, but not with DAQA-C8, was unclear.

For the two-layer approach, the first layer, DA, has led to a thick deposit, with a 13% increase in C-O/C-N (Table 3), suggesting that a significant amount of dopamine or polydopamine was formed on the surface. Further immersion of the DAQA-Cx solution in the second step led to an increase in C-O/C-N, indicating successful deposition of DAQA-Cx on the DA-coated Ti substrate. Nevertheless, the C-O/C-N area percentage did not vary significantly with the DAQA-Cx concentration. In contrast to the single-layer approach, all two-layer DAQA-Cx-modified samples have led to the disappearance of Ti-C binding.

### 3.3. Antibacterial Assay

**Figure 5:**
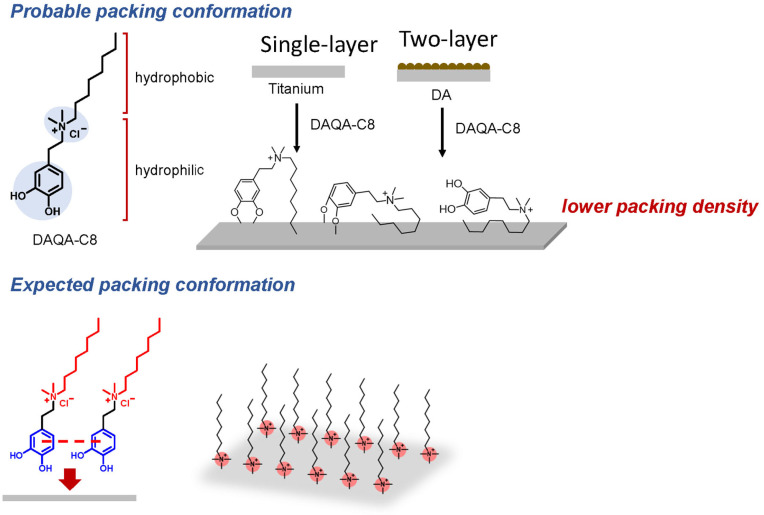
The schematic drawing of the likely packing for the DAQA-C8 on the Ti (single-layer approach) or DA (two-layer approach) substrate. The dotted line representing the interactions between the DAQA-C8. The arrow showed the interaction/deposition between the DAQA-C8 and the Ti substrate.

The antibacterial activity of different modified titanium substrates was first examined against Gram-negative *E. coli* (ATCC 23501) using bacterial reduction percentage (Table 4). For substrates modified by the first-layer approach, those modified with DAQA-C8 exhibited negative bacterial reduction percentages; i.e., more bacteria than on the bare, non-modified Ti. On the other hand, the ones modified by the compounds with the shorter alkyl chain, DAQA-C4 and DAQA-C1, exhibited various antibacterial activities against *E. coli*. In contrast to some studies [33,36,37] and our earlier study [2] that have indicated that longer alkyl chains enhance the antibacterial activity of surfaces grafted/immobilized with a cationic quaternary ammonium “polymeric” chain, this study showed higher antimicrobial activity with shorter alkyl chains for the “small” cationic molecules when the same immersion concentration was used. It is speculated that the hydrophilic surface prepared using small cationic molecules here, without the waving polymeric brushes as shown in these studies [2,33,36,37], would be easier for microbes migrating from the buffer to direct contact with the cationic ends on the surface (i.e., no brush steric hindrance effect), leading to a higher bactericidal effect. On the other hand, for surfaces prepared with cationic quaternary ammonium “polymeric” brushes, microbes would contact the cationic groups at the brush ends, leading to bacterial death. A longer alkyl chain associated with the cationic terminal ends of these polymeric brushes could enhance the interactions between the adhered microbes and cationic functionalities. Furthermore, for these polymeric-brush-modified surfaces, a higher grafting density of cationic polymeric brushes would also lead to a greater antibacterial activity [2,33,36,37]. Combining the antibacterial activity, surface hydrophilicity, and XPS C4N^+^ percentage findings, we speculate that the immobilized density of DAQA-Cx on the Ti substrate may not be high enough to force the DAQA-Cx chain orientation away from the Ti substrate surface plane, as suggested by theory on self-assembled monolayers (SAMs) [49,50]. Rather, the immobilized DAQA-Cx chain was speculated to lie along the Ti surface plane (top panel in Figure 5) rather than the lower panel in Figure 5 or the Scheme shown in Figure 2.

For the two-layer approach, the first layer, the dopamine-coated layer DA, exhibited slightly antibacterial capability, <40%. After deposition of a second layer of cationic compounds with different alkyl chain lengths, the two-layer-modified Ti substrates exhibited a similar antibacterial trend to those modified by the single-layer approach, with antibacterial activity decreasing with increasing alkyl chain length at the same immersion concentration. This could be attributed to the same cause: surface hydrophilicity would enable more contact between the surface cationic quaternary ammonium functionality and *E. coli*, resulting in higher antibacterial activity. The highest antibacterial activity against *E. coli* was observed in DA_C1-2 and DA_C1-8 (~88–95% bacterial reduction) since both exhibited the lowest contact angles (Figure 4b). Again, based on the XPS data about C_4_N^+^ variation with the alkyl chain length (Table 2), bacterial reduction (Table 4), and the contact angle results (Figure 4b), we speculate that the chain orientation of the DAQA-Cx on the first dopamine-coated layer would be similar to that on the Ti surface by the single-layer approach (Figure 5). Nevertheless, further analyses would be needed to elucidate these findings across the single-layer and two-layer approaches and to examine the likely mechanistic models.

Since the surfaces modified by the single-layer approach showed low antibacterial activity against *E. coli*, only those modified by the two-layer approach were further examined for their antibacterial activity against *S. aureus*. In contrast to the antibacterial activity against *E. coli*, most of the two-layer-modified surfaces, except DA_C1-8, showed less than 41.6% or even no antibacterial activity against *S. aureus* (Table 4).

The discrepancy in antibacterial activity between Gram-negative *E. coli* (ATCC 23501) and Gram-positive *S. aureus* (ATCC 21351) stems from differences in their cell wall structures [51]. Although *S. aureus* has only one phospholipid layer, the peptidoglycan layer covering the inner phospholipid structure is thicker than that of *E. coli*. The peptidoglycan layer of Gram-negative *E. coli* is 3–6 nm thick, while the cell wall thickness of Gram-positive *S. aureus* is 5–10 times that of *E. coli*, and its peptidoglycan layer is about 34 nm thick [52]. The peptidoglycan layer mainly can support and stabilize the entire bacterial endoplasm, maintain the shape of the bacterial cell and prevent the peptidoglycan from expanding and rupturing due to the difference in osmotic pressure inside and outside the cell [53]. Previous studies on the cationic “polymer” with quaternary ammonium functionality, including our earlier study on the catechol-terminated “polymeric” quaternary ammonium functional ends [3,18], have shown a similar or slightly better antibacterial activity against Gram-positive *S. aureus* than the Gram-negative *E. coli*. On the other hand, as in our current study using cationic “small” molecules, better antibacterial activity against *E. coli* than against *S. aureus* was observed when the cationic quaternary ammonium “small” molecule was used to modify the substrate [54]. This is because small molecules do not penetrate the thick peptidoglycan layer of *S. aureus* as polymers do. The bactericidal small molecules are almost all located outside the peptidoglycan layer [55] and do not penetrate it. They attract the negatively charged phospholipid bilayer to the surface by the positive charge on the surface. Furthermore, the peptidoglycan layer may slow down the rate at which the inner negatively charged phospholipid layer dissociates to the positively charged surface, potentially making bacteria less susceptible to death. *E. coli* has a thinner peptidoglycan layer but an additional phospholipid layer; given a high enough surface charge density, more and faster negatively charged phospholipids would be dissociated, leading to microbial death. Furthermore, the peptidoglycan layer of *E. coli* is even thinner than that of *S. aureus*, making it easier for the inner negatively charged phospholipid structure of *E. coli* to dissociate. Henceforth, antibacterial activity against *E. coli* is higher than against *S. aureus* for small bactericidal cationic molecules, such as those reported in this study.

## 4. Conclusions

Three novel catechol-terminated compounds with quaternary ammonium functionality at different alkyl chain lengths, namely DAQA-C8, DAQA-C4, and DAQA-C1, were synthesized with high purity at a yield rate greater than 65%. Contact angle analysis revealed that the two-sided coating method used here was effective at creating a uniform coating on both front and back surfaces, with minimal variation. Further, the surface hydrophobicity increased with alkyl chain length in both the direct single-layer coating and the two-layer method, in which the dopamine first layer was formed before immersion in the cationic quaternary ammonium compound.

XPS analyses revealed that the deposit formed by the direct single-layer method was very thin. In contrast, with the two-layer approach, the deposit thickness would greatly increase, and the surface cationic quaternary ammonium percentage would be higher than that of the single-layer counterpart. Either in the surfaces modified by the single-layer or two-layer technique, the highest cationic quaternary ammonium percentage was noted on the one prepared by the longest alkyl chain, DAQA-C8.

The highest antibacterial activity against Gram-negative *E. coli* was observed on surfaces prepared using the two-layer approach with the cationic quaternary ammonium compounds having the shortest alkyl chains, DA_C1-2 and DA_C1-8. This is likely due to the synergistic effect of the surface cationic quaternary ammonium percentage and surface hydrophilicity, which enhance microbial contact with the bactericidal surface cationic functionalities. Nevertheless, the antibacterial activity against Gram-positive *S. aureus* was significantly reduced, likely due to the thicker peptidoglycan layer and cell wall thickness associated with *S. aureus*.

In summary, this study has demonstrated that the novel small molecule with a catechol terminal and cationic quaternary ammonium functionality with different alkyl chain lengths can be effectively used for antibacterial coating on medical-grade titanium substrates against Gram-negative *E. coli* using the two-layer approach. Nevertheless, a polymeric catechol-terminated compound with cationic quaternary ammonium functionalities, such as the PQA-C8 reported earlier by our group [3], would be an alternative for an effective antibacterial coating on medical-grade titanium against Gram-positive *S. aureus*. For medical plastic devices, further studies are underway to explore the potential of using these small molecules as antibacterial coatings. Furthermore, evaluating the durability and retention of antimicrobial performance after multiple cleaning/sterilization cycles, which are commonly encountered in clinical settings, for these modified titanium substrates should be undertaken to ensure their use in clinical settings.

## Figures and Tables

**Figure 3:**
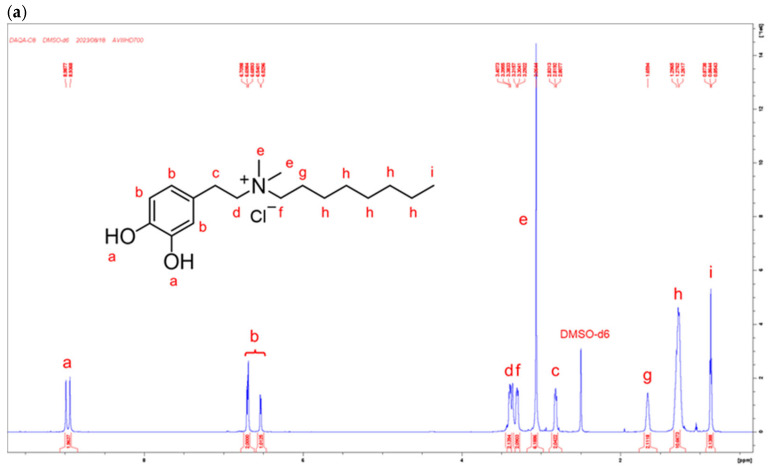

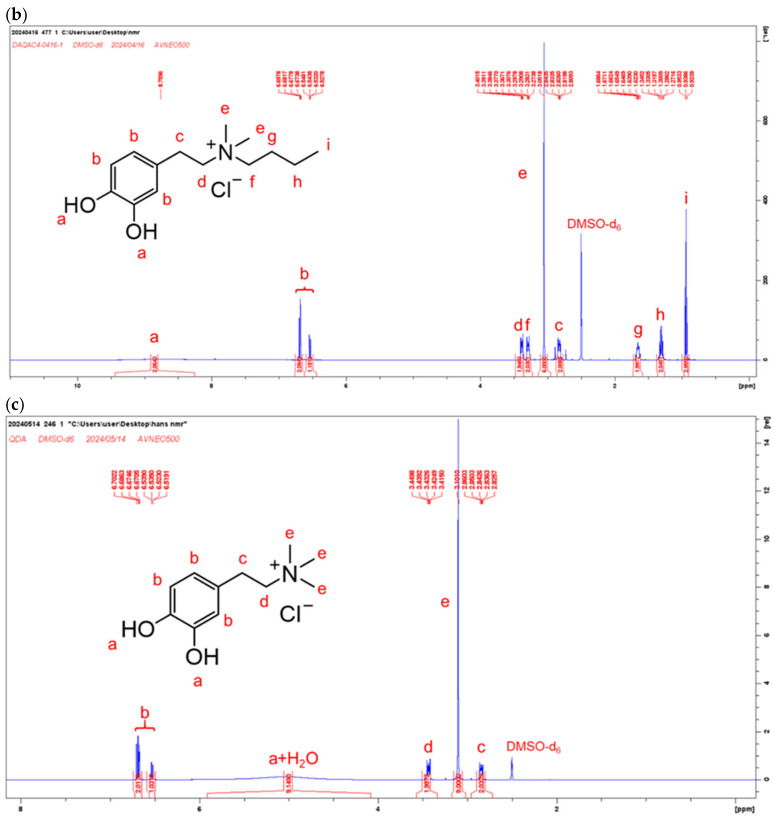
The ^1^H-NMR spectra of (**a**) DAQA-C8 (700 MHz, DMSO-d6) δ (ppm): 8.99–8.93 (d, 2H), 6.71–6.68 (m, 2H), 6.55–6.52 (m, 1H), 3.41–3.38 (m, 2H), 3.32–3.29 (m, 2H), 3.10–3.02 (s, 6H), 2.84–2.80 (m, 2H), 1.72–1.58 (m, 2H), 1.45–1.15 (m, 10H), 0.88–0.85 (t, 3H). (**b**) DAQA-C4 (500 MHz, DMSO-d6) δ (ppm): 9.45–8.25 (s, 2H), 6.70–6.67 (m, 2H), 6.55–6.52 (m, 1H), 3.41–3.36 (m, 2H), 3.31–3.27 (m, 2H), 3.12–2.98 (s, 6H), 2.85–2.80 (m, 2H), 1.69–1.62 (m, 2H), 1.35–1.27 (m, 2H), 0.96–0.92 (t, 3H). (**c**) DAQA-C1 (500 MHz, DMSO-d6) δ (ppm): 9.45–8.25 (s, 2H), 6.71–6.67 (m, 2H), 6.54–6.51 (m, 1H), 3.45–3.41 (m, 2H), 3.15–3.07 (s, 6H), 2.87–2.82 (m, 2H).

**Table 1:** Sample nomenclature for the samples modified by different protocols.

Sample Name	Treatment Scheme
bare Ti	Ti substrate was cleaned without Piranha solution immersion
Ti-OH	bare Ti was immersed in Piranha solution for 1 h
Single-layer approach	
C8-x	Ti-OH was coated with x mg/mL DAQA-C8 for 24 h; x = 0.5, 2, and 8
C4-x	Ti-OH was coated with x mg/mL DAQA-C4 for 24 h; x = 0.5, 2, and 8
C1-x	Ti-OH was coated with x mg/mL DAQA-C1 for 24 h; x = 0.5, 2, and 8
Two-layer approach	
DA	Ti-OH was coated with 2 mg/mL dopamine for 4 h as the first layer
DA_C8-x	DA was further coated with x mg/mL of DAQA-C8 for 24 h; x = 0.5, 2, and 8
DA_C4-x	DA was further coated with x mg/mL of DAQA-C4 for 24 h; x = 0.5, 2, and 8
DA_C1-x	DA was further coated with x mg/mL of DAQA-C1 for 24 h; x = 0.5, 2, and 8

**Table 2:** The surface atomic percentages of various Ti substrates modified by the single-layer and two-layer approaches.

Sample	Ti2p	C1s	N1s	O1s	C_4_N^+^	C_4_N^+^/N1s
bare Ti	17.7%	38.0%	2.4%	41.8%	0%	0
Ti-OH	17.7%	29.7%	0.8%	51.8%	0%	0
Single-layer approach						
C8-0.5	17.5%	41.5%	1.9%	39.1%	0.70%	0.37
C8-2	10.0%	51.4%	2.5%	36.1%	0.91%	0.36
C8-8	0.2%	75.6%	3.8%	20.4%	2.70%	0.71
C4-0.5	16.7%	36.1%	0.9%	46.3%	0.15%	0.17
C4-2	15.6%	37.5%	1.0%	45.9%	0.19%	0.19
C4-8	14.9%	39.3%	1.4%	44.5%	0.34%	0.24
C1-0.5	15.7%	36.8%	1.3%	46.3%	0.29%	0.22
C1-2	15.4%	38.1%	1.3%	45.1%	0.48%	0.37
C1-8	15.6%	38.7%	1.5%	44.2%	0.56%	0.37
Two-layer approach						
DA	0%	70.1%	7.2%	22.7%	0%	0
DA_C8-0.5	0%	75.2%	5.4%	19.4%	1.16%	0.21
DA_C8-2	0%	77.1%	4.2%	18.7%	1.44%	0.34
DA_C8-8	0%	76.8%	3.7%	19.5%	2.64%	0.71
DA_C4-0.5	0%	70.1%	6.4%	23.5%	0.87%	0.14
DA_C4-2	0.1%	70.5%	6.0%	23.4%	1.31%	0.22
DA_C4-8	0.2%	71.8%	5.6%	22.5%	1.50%	0.27
DA_C1-0.5	0%	70.6%	6.2%	23.2%	1.02%	0.16
DA_C1-2	0%	70.3%	6.0%	23.7%	1.60%	0.27
DA_C1-8	0%	70.5%	6.0%	23.5%	1.74%	0.29

**Table 3:** The C1s area percentages of the deconvoluted peaks for various Ti substrates modified by the single-layer and two-layer approaches.

Sample	C-C/C-H/C=C (285 eV)	C-O/C-N (286.6 eV)	C=O (288.6 eV)	Ti-C (282 eV)
bare Ti	63.4%	8.0%	17.1%	11.5%
Ti-OH	72.1%	13.7%	14.2%	0%
Single-layer approach				
C8-0.5	70.7%	18.6%	10.0%	0.7%
C8-2	67.6%	24.0%	8.4%	0%
C8-8	62.7%	31.8%	5.5%	0%
C4-0.5	72.2%	17.1%	9.0%	1.7%
C4-2	72.1%	17.3%	10.0%	0.5%
C4-8	66.8%	21.5%	10.5%	1.1%
C1-0.5	70.1%	17.8%	10.9%	1.3%
C1-2	73.5%	16.1%	9.6%	0.8%
C1-8	71.8%	17.9%	9.2%	1.1%
Two-layer approach				
DA	66.5%	26.8%	6.7%	0%
DA_C8-0.5	60.5%	32.1%	7.3%	0%
DA_C8-2	63.9%	30.5%	5.6%	0%
DA_C8-8	63.8%	31.2%	5.1%	0%
DA_C4-0.5	57.9%	33.9%	8.2%	0%
DA_C4-2	56.8%	32.4%	10.9%	0%
DA_C4-8	57.8%	34.6%	7.6%	0%
DA_C1-0.5	54.9%	36.6%	8.5%	0%
DA_C1-2	53.3%	37.9%	8.8%	0%
DA_C1-8	54.0%	37.7%	8.3%	0%

**Table 4:** The bacterial reduction percentages for various Ti substrates modified by the single-layer and two-layer approaches against *E. coli* and *S. aureus*.

Sample	Bacterial Reduction (%)	
	*E. coli* (ATCC 23501)	*S. aureus* (ATCC 21351)
	Single-layer approach	
C8-0.5	*	n.d.
C8-2	*	n.d.
C8-8	*	n.d.
C4-0.5	71.6 ± 22.2	n.d.
C4-2	<40	n.d.
C4-8	<40	n.d.
C1-0.5	76.1 ± 22.1	n.d.
C1-2	57.8 ± 25.6	n.d.
C1-8	73.4 ± 20.3	n.d.
	Two-layer approach	
DA	<40	*
DA_C8-0.5	*	*
DA_C8-2	*	<40
DA_C8-8	*	<40
DA_C4-0.5	<30	<40
DA_C4-2	79.4 ± 15.8	<40
DA_C4-8	<30	<40
DA_C1-0.5	<50	<40
DA_C1-2	90.6 ± 5.9	41.6 ± 4.9
DA_C1-8	88.3 ± 7.3	60.7 ± 22.7

*: More bacteria were noted as compared to the non-modified control, bare Ti; i.e., negative antibacterial reduction percentage. n.d.: not determined.

## Data Availability

The original contributions presented in this study are included in the article/Supplementary Materials. Further inquiries can be directed to the corresponding author.

## Supplementary Materials

The following supporting information can be downloaded at: https://www.mdpi.com/article/10.3390/jfb17060271/s1, Figure S1. The ^1^H-NMR spectra of TFADA (700 MHz, DMSO-d_6_) δ (ppm): 9.45–9.42 (t, 1H), 8.76–8.67 (d, 2H), 6.64–6.62 (d, 1H), 6.58–6.57 (d, 1H), 6.44–6.42 (d, 1H), 3.33–3.28 (q, 2H), 2.62–2.59 (t, 2H). Figure S2. The ^1^H-NMR spectra of TFADAAC (700 MHz, CDCl_3_) δ (ppm): 6.68–6.57 (m, 3H), 6.37–6.18 (s, 1H), 3.60–3.55 (q, 2H), 2.79–2.76 (t, 2H), 1.69–1.65 (s, 6H). Figure S3. The ^1^H-NMR spectra of DAAC (700 MHz, CDCl_3_) δ (ppm): 6.61–6.59 (m, 1H), 6.56–6.54 (m, 2H), 2.88–2.85 (t, 2H), 2.61–2.59 (t, 2H), 1.65–1.58 (s, 6H), 1.56–1.47 (s, 2H)in CDCl_3_. Figure S4. The ^1^H-NMR spectra of OADAAC (500 MHz, CDCl_3_) δ (ppm): 6.66–6.63 (m, 1H), 6.58–6.56 (m, 2H), 5.50–5.35 (s, 1H), 3.48–3.43 (q, 2H), 2.72–2.68 (t, 2H), 2.14–2.09 (t, 2H), 1.68–1.64 (s, 6H), 1.62–1.55 (m, 2H), 1.35–1.18 (m, 8H), 0.89–0.85 (t, 3H). Figure S5. The ^1^H-NMR spectra of ROADAAC (700 MHz, CDCl_3_) δ (ppm): 6.64–6.62 (m, 1H), 6.60–6.58 (m, 2H), 2.82–2.79 (t, 2H), 2.71–2.68 (t, 2H), 2.60–2.57 (t, 2H), 1.67–1.63 (s, 6H), 1.48–1.42 (m, 2H), 1.30–1.20 (m, 11H), 0.88–0.85 (t, 3H). Figure S6. The ^1^H-NMR spectra of DAACQA-C8 (700 MHz, CDCl_3_) δ (ppm): 6.74–6.70 (m, 2H), 6.64–6.62 (m, 1H), 3.71–3.67 (m, 2H), 3.58–3.54 (m, 2H), 3.44–3.41 (s, 6H), 3.01–2.98 (m, 2H), 1.71–1.66 (m, 2H), 1.63–1.60 (s, 6H), 1.36–1.19 (m, 10H), 0.86–0.83 (t, 3H). Figure S7. The ^1^H-NMR spectra of BADAAC (500 MHz, CDCl_3_) δ (ppm): 6.66–6.63 (m, 1H), 6.59–6.56 (m, 2H), 5.65–5.35 (s, 1H), 3.48–3.43 (q, 2H), 2.72–2.68 (t, 2H), 2.13–2.09 (t, 2H), 1.68–1.64 (s, 6H), 1.65–1.58 (m, 2H), 0.94–0.90 (t, 3H). Figure S8. The ^1^H-NMR spectra of RBADAAC (500 MHz, CDCl_3_) δ (ppm): 6.64–6.62 (m, 1H), 6.60–6.58 (m, 2H), 2.82–2.78 (t, 2H), 2.70–2.66 (t, 2H), 2.60–2.56 (t, 2H), 1.80–1.40 (s, 6H), 1.80–1.40 (s, 1H), 1.46–1.40 (m, 2H), 1.33–1.25 (m, 2H), 0.89–0.85 (t, 3H). Figure S9. The ^1^H-NMR spectra of DAACQA-C4 (500 MHz, CDCl_3_) δ (ppm): 6.76–6.72 (m, 2H), 6.65–6.62 (m, 1H), 3.71–3.67 (m, 2H), 3.63–3.59 (m, 2H), 3.45–3.35 (s, 6H), 3.04–3.00 (m, 2H), 1.74–1.67 (m, 2H), 1.65–1.56 (s, 6H), 1.45–1.37 (m, 2H), 0.99–0.95 (t, 3H). Figure S10. The ^1^H-NMR spectra of FADAAC (500 MHz, CDCl_3_) δ (ppm): 8.23–8.12 (s, 1H), 6.66–6.63 (m, 1H), 6.60–6.58 (m, 2H), 6.10–6.38 (s, 1H), 3.55–3.50 (q, 2H), 2.76–2.73 (t, 2H), 1.70–1.62 (s, 6H). Figure S11. The ^1^H-NMR spectra of RFADAAC (500 MHz, CDCl_3_) δ (ppm): 6.61–6.59 (m, 1H), 6.57–6.55 (m, 2H), 2.78–2.74 (t, 2H), 2.69–2.65 (t, 2H), 2.43–2.36 (s, 3H), 1.86–1.66 (s, 1H), 1.64–1.59 (s, 6H) in CDCl_3_. Figure S12. The ^1^H-NMR spectra of QDAAC (DAACQA-C1) (500 MHz, CDCl_3_) δ (ppm): 6.76–6.72 (m, 2H), 6.67–6.65 (m, 1H), 3.81–3.77 (m, 2H), 3.57–3.44 (s, 9H), 3.06–3.02 (m, 2H), 1.72–1.60 (s, 6H). Figure S13. The C1S deconvolution spectra for different titanium substrates prepared by the single-layer and two-layer approaches.
