# Supplementary material for: Surface Characterization and Antimicrobial Capability Evaluation of Medical-Grade Titanium Modified by Facile Immersion in the Solution of Novel Catechol-Terminated Compounds Having Cationic Quaternary Ammonium Functionality with Different Alkyl Chain Lengths
Source: J Funct Biomater. 2026 Jun 1;17(6):271. doi: 10.3390/jfb17060271 (PMC13301612; doi:10.3390/jfb17060271)
Supplement: Supplementary file 1 [file jfb-17-00271-s001.zip › jfb-4305295-supplementary.pdf]

Surface characterization and antimicrobial capability evaluation of medical-grade titanium modified by facile immersion in the solution of novel catechol-terminated compounds having cationic quaternary ammonium functionality with different alkyl chain lengths

Zong-Hua Liu[1], Nai-Chia Fan[2,3], Chi-Hui Cheng[2,4], Jui-Che Lin[1,5*]

[1] Department of Chemical Engineering, National Cheng Kung University, Tainan, Taiwan

[2] Division of Nephrology, Department of Pediatrics, Chang Gung Memorial Hospital, Taoyuan, Taiwan

[3] Graduate Institute of Clinical Medical Sciences, Chang Gung University, Taoyuan, Taiwan

[4] Department of Pediatrics, College of Medicine, Chang Gung University, Taoyuan, Taiwan

[5] School of Dentistry, Institute of Oral Medicine, College of Medicine, National Cheng Kung University, Tainan, Taiwan

Corresponding Author

* Prof. Jui-Che Lin: Department of Chemical Engineering, National Cheng Kung University, Tainan, TAIWAN 70101, Phone: +886-6-275-7575 ext. 62665, Fax: +886-6-234-4996, Email: jclin@mail.ncku.edu.tw ORCID ID: https://orcid.org/0000-0003-4436-655X

3. Results and Discussion

3.1 The NMR spectra of the intermediates for synthesizing the DAQA-C8, DAQA-C4, and DAQA-C1

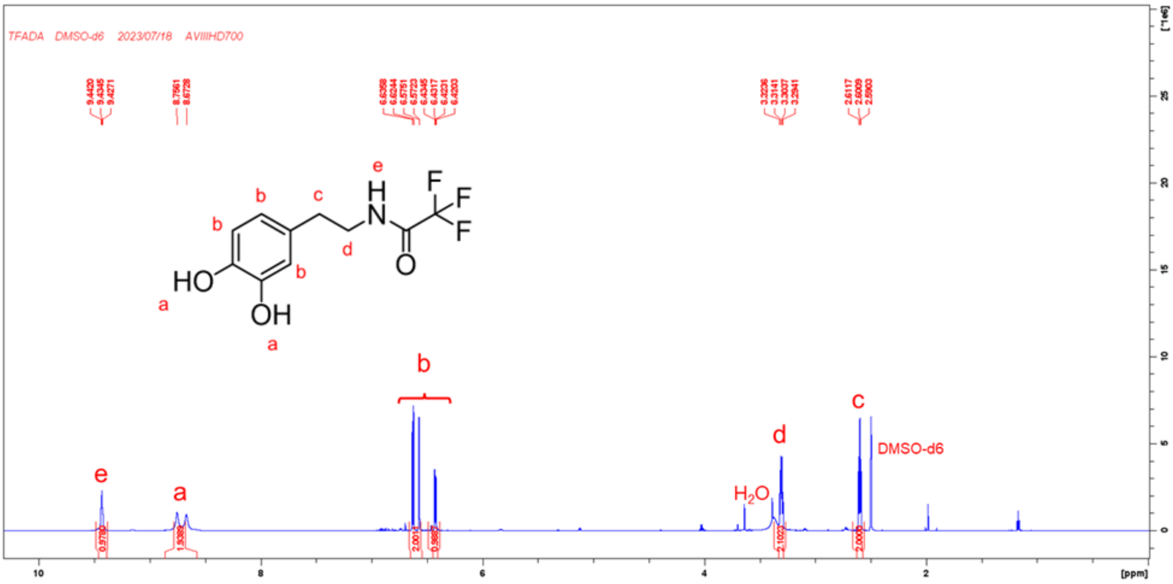

Figure S1 The ^{1}H-NMR spectra of TFADA (700 MHz, DMSO-d_6) δ (ppm): 9.45 - 9.42 (t, 1H), 8.76 - 8.67 (d, 2H), 6.64 -6.62 (d, 1H), 6.58 - 6.57 (d, 1H), 6.44 - 6.42 (d, 1H), 3.33 - 3.28 (q, 2H), 2.62 – 2.59 (t, 2H)

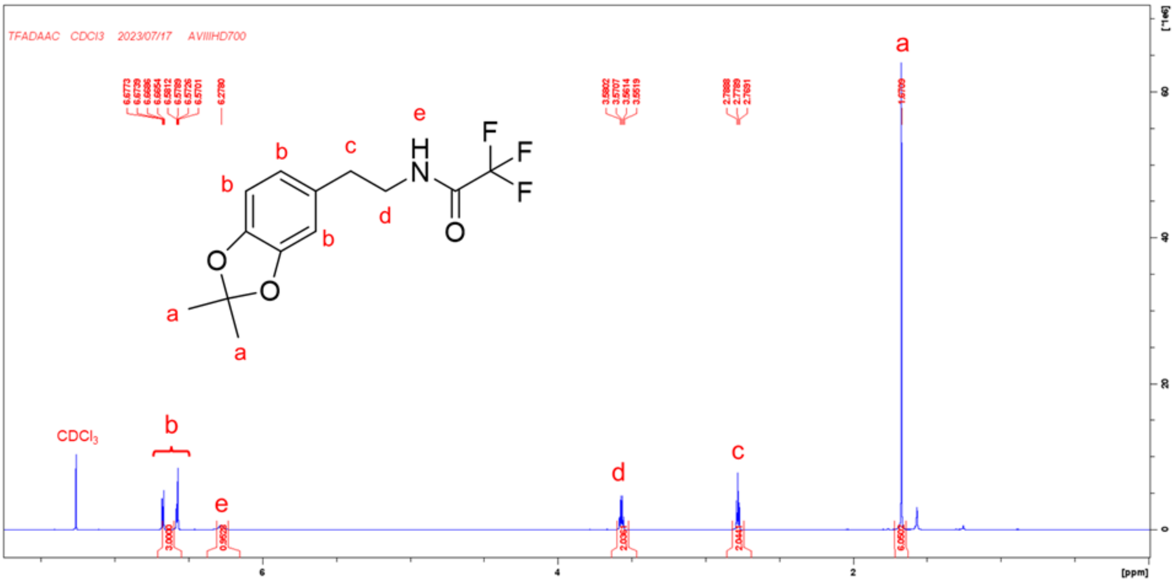

Figure S2 The ^{1}H-NMR spectra of TFADAAC (700 MHz, $CDCl_3$) δ (ppm): 6.68 - 6.57 (m, 3H), 6.37 - 6.18 (s, 1H), 3.60 -3.55 (q, 2H), 2.79 - 2.76 (t, 2H), 1.69 - 1.65 (s, 6H)

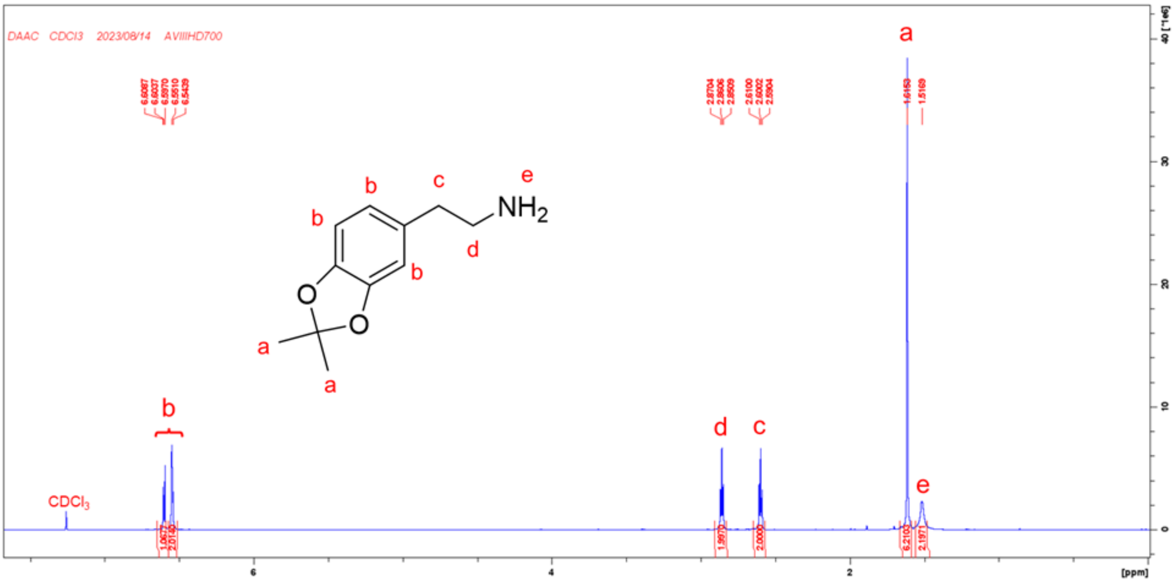

Figure S3 The ^{1}H-NMR spectra of DAAC (700 MHz, $CDCl_3$) δ (ppm): 6.61 - 6.59 (m, 1H), 6.56 - 6.54 (m, 2H), 2.88 -2.85 (t, 2H), 2.61 - 2.59 (t, 2H), 1.65 - 1.58 (s, 6H), 1.56 - 1.47 (s, 2H)in $CDCl_3$

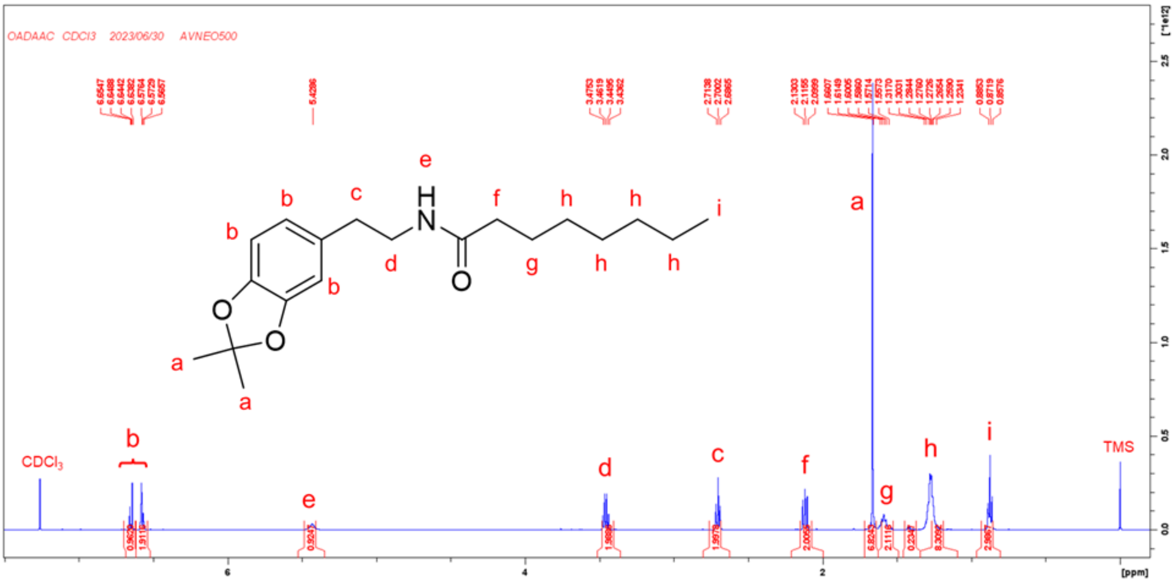

Figure S4 The ^{1}H-NMR spectra of OADAAC (500 MHz, $CDCl_3$) δ (ppm): 6.66 - 6.63 (m, 1H), 6.58 - 6.56 (m, 2H), 5.50 -5.35 (s, 1H), 3.48 - 3.43 (q, 2H), 2.72 - 2.68 (t, 2H), 2.14 - 2.09 (t, 2H), 1.68 - 1.64 (s, 6H), 1.62 - 1.55 (m, 2H), 1.35 - 1.18 (m, 8H), 0.89 - 0.85 (t, 3H)

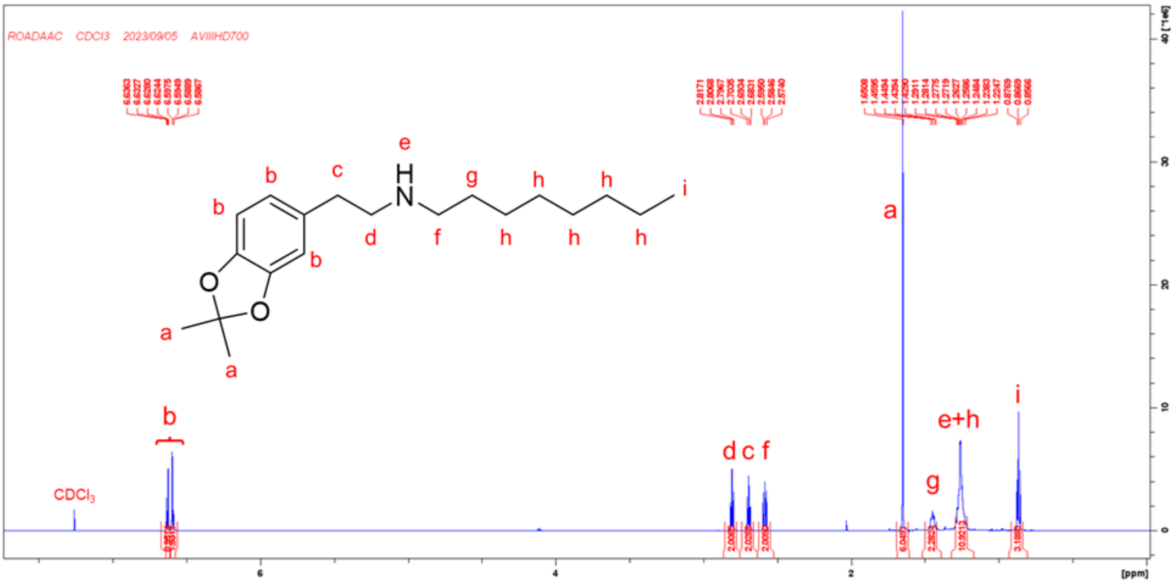

Figure S5 The ^{1}H-NMR spectra of ROADAAC (700 MHz, $CDCl_3$) δ (ppm): 6.64 - 6.62 (m, 1H), 6.60 - 6.58 (m, 2H), 2.82 - 2.79 (t, 2H), 2.71 - 2.68 (t, 2H), 2.60 - 2.57 (t, 2H), 1.67 - 1.63 (s, 6H), 1.48 - 1.42 (m, 2H), 1.30 - 1.20 (m, 11H), 0.88 - 0.85 (t, 3H)

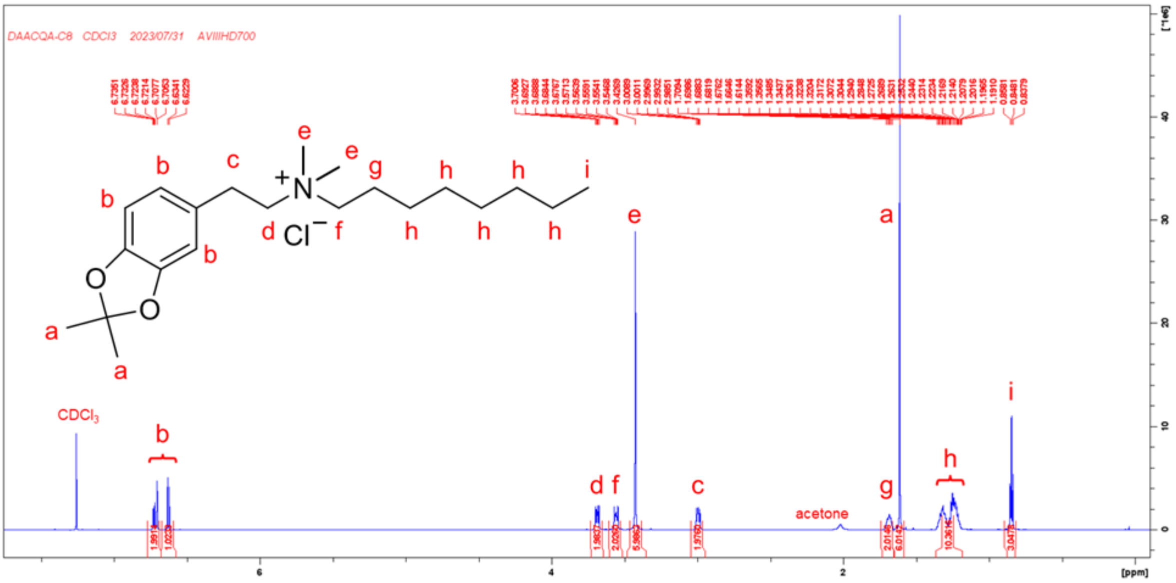

Figure S6 The ^{1}H-NMR spectra of DAACQA-C8 (700 MHz, $CDCl_3$) δ (ppm): 6.74 - 6.70 (m, 2H), 6.64 - 6.62 (m, 1H), 3.71 - 3.67 (m, 2H), 3.58 - 3.54 (m, 2H), 3.44 - 3.41 (s, 6H), 3.01 - 2.98 (m, 2H), 1.71 - 1.66 (m, 2H), 1.63 - 1.60 (s, 6H), 1.36 - 1.19 (m, 10H), 0.86 - 0.83 (t, 3H)

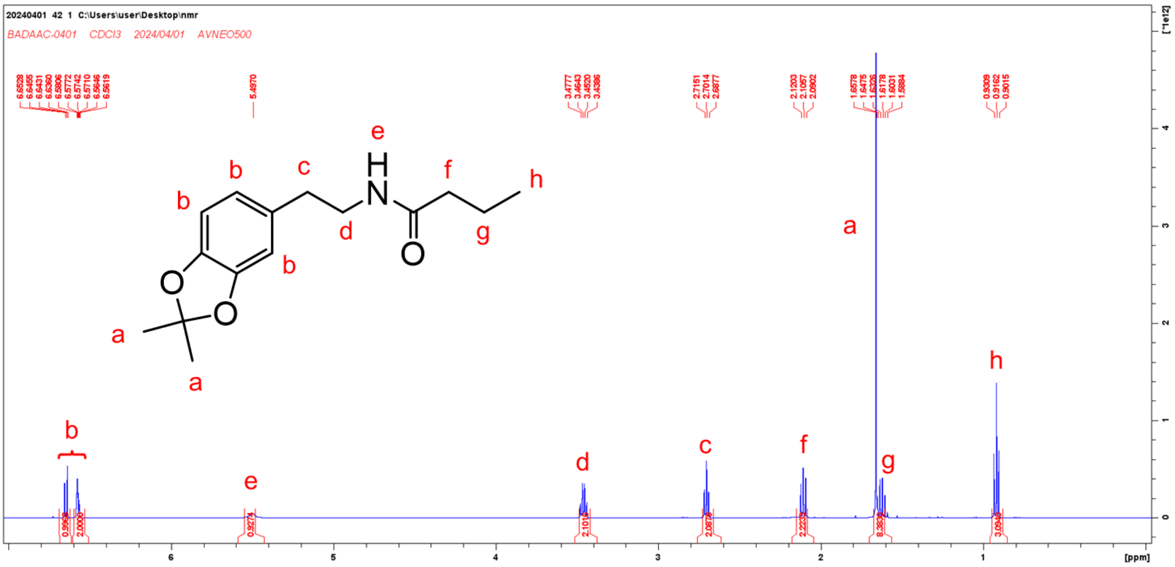

Figure S7 The ^{1}H-NMR spectra of BADAAC (500 MHz, $CDCl_3$) δ (ppm): 6.66 - 6.63 (m, 1H), 6.59 - 6.56 (m, 2H), 5.65 -5.35 (s, 1H), 3.48 - 3.43 (q, 2H), 2.72 - 2.68 (t, 2H), 2.13 - 2.09 (t, 2H), 1.68 - 1.64 (s, 6H), 1.65 - 1.58 (m, 2H), 0.94 - 0.90 (t, 3H)

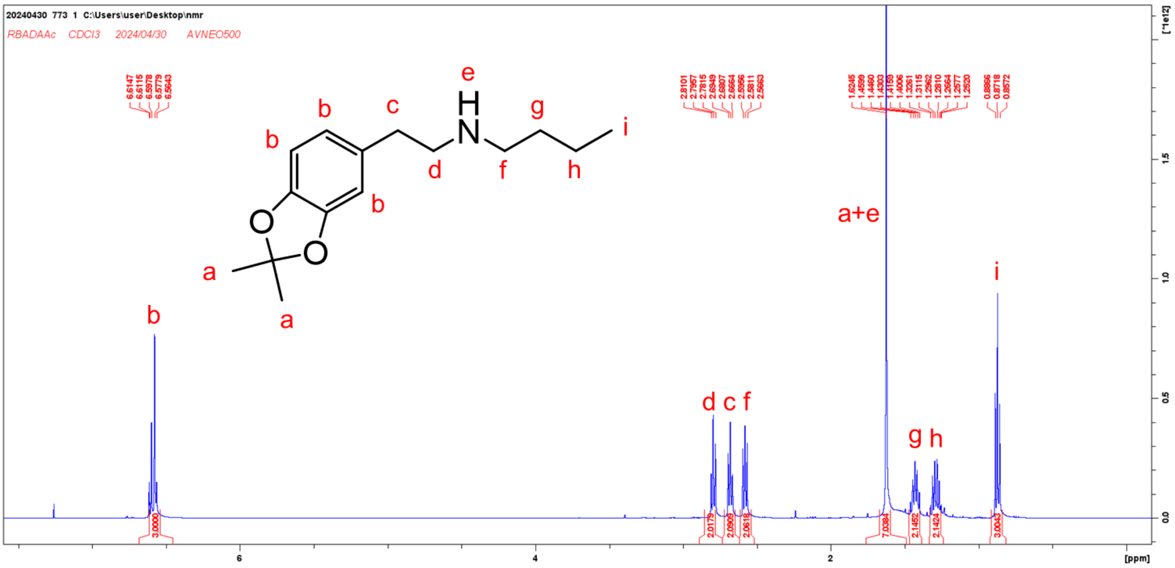

Figure S8 The ^{1}H-NMR spectra of RBADAAC (500 MHz, $CDCl_3$) δ (ppm): 6.64 - 6.62 (m, 1H), 6.60 - 6.58 (m, 2H), 2.82 - 2.78 (t, 2H), 2.70 - 2.66 (t, 2H), 2.60 - 2.56 (t, 2H), 1.80 - 1.40 (s, 6H), 1.80 - 1.40 (s, 1H), 1.46 - 1.40 (m, 2H), 1.33 - 1.25 (m, 2H), 0.89 – 0.85 (t, 3H)

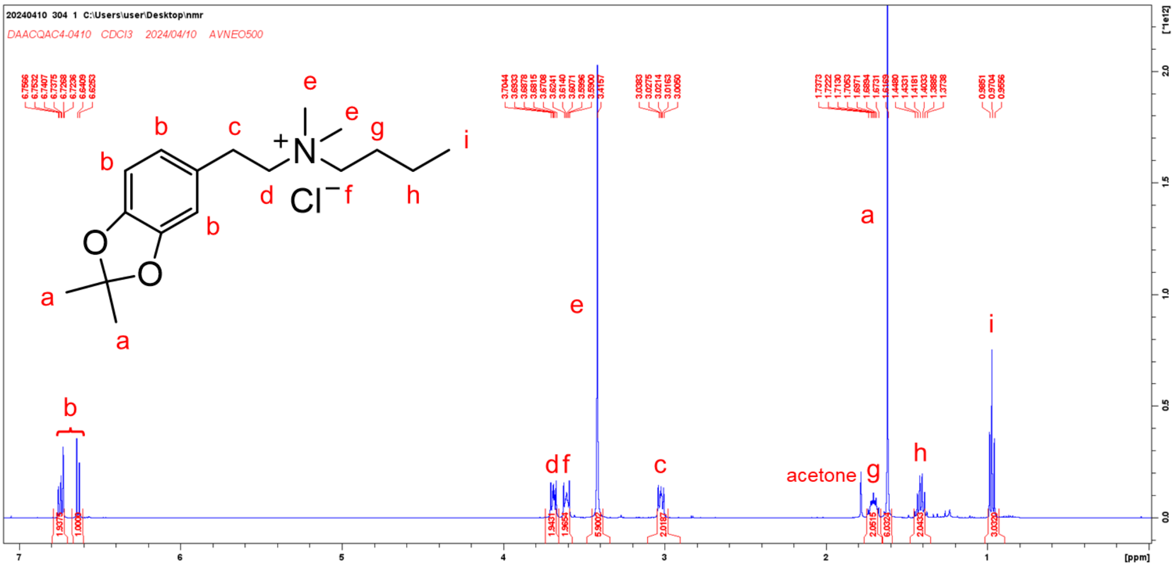

Figure S9 The ^{1}H-NMR spectra of DAACQA-C4 (500 MHz, $CDCl_3$) δ (ppm): 6.76 - 6.72 (m, 2H), 6.65 - 6.62 (m, 1H), 3.71 - 3.67 (m, 2H), 3.63 - 3.59 (m, 2H), 3.45 - 3.35 (s, 6H), 3.04 - 3.00 (m, 2H), 1.74 - 1.67 (m, 2H), 1.65 - 1.56 (s, 6H), 1.45 - 1.37 (m, 2H), 0.99 - 0.95 (t, 3H)

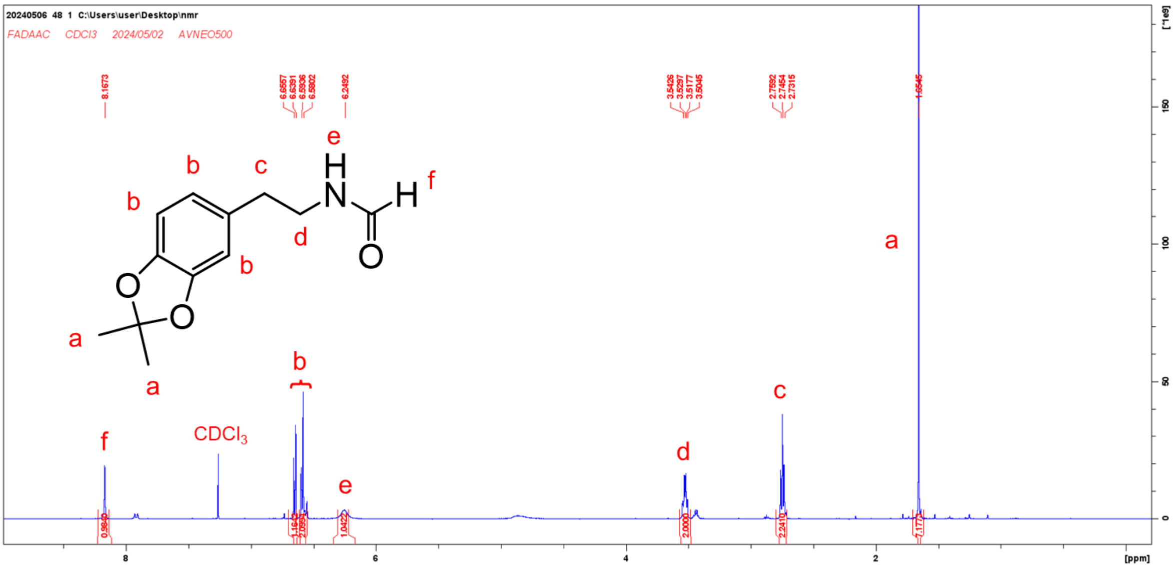

Figure S10 The ^{1}H-NMR spectra of FADAAC (500 MHz, $CDCl_3$) δ (ppm): 8.23 - 8.12 (s, 1H), 6.66 - 6.63 (m, 1H), 6.60 - 6.58 (m, 2H), 6.10 - 6.38 (s, 1H), 3.55 - 3.50 (q, 2H), 2.76 - 2.73 (t, 2H), 1.70 - 1.62 (s, 6H)

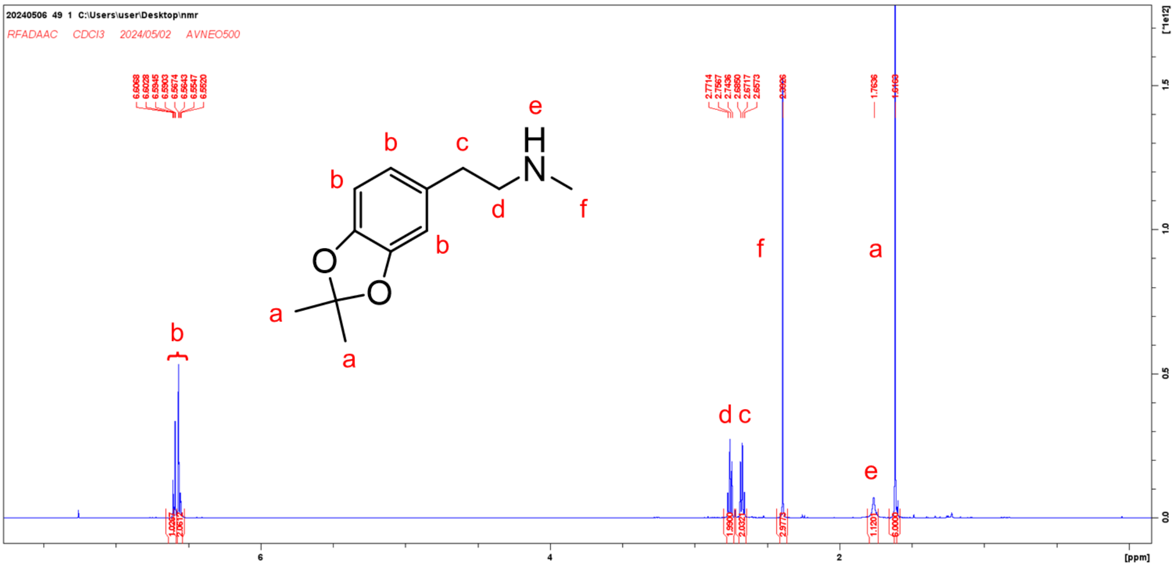

Figure S11 The ^{1}H-NMR spectra of RFADAAC (500 MHz, $CDCl_3$) δ (ppm): 6.61 - 6.59 (m, 1H), 6.57 - 6.55 (m, 2H), 2.78 - 2.74 (t, 2H), 2.69 - 2.65 (t, 2H), 2.43 - 2.36 (s, 3H), 1.86 - 1.66 (s, 1H), 1.64 - 1.59 (s, 6H) in $CDCl_3$

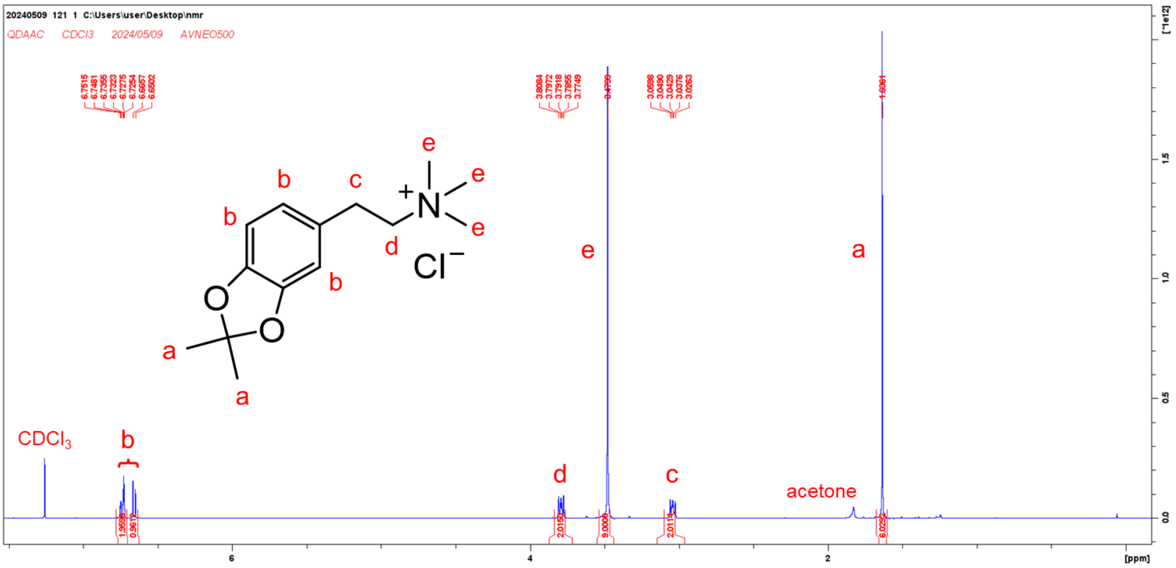

Figure S12 The ^{1}H-NMR spectra of QDAAC (DAACQA-C1) (500 MHz, $CDCl_3$) δ (ppm): 6.76 - 6.72 (m, 2H), 6.67 - 6.65 (m, 1H), 3.81 - 3.77 (m, 2H), 3.57 - 3.44 (s, 9H), 3.06 - 3.02 (m, 2H), 1.72 - 1.60 (s, 6H)

3.2 Curve fitting for the C1s of the surfaces modified by the single-layer and double-layer approach

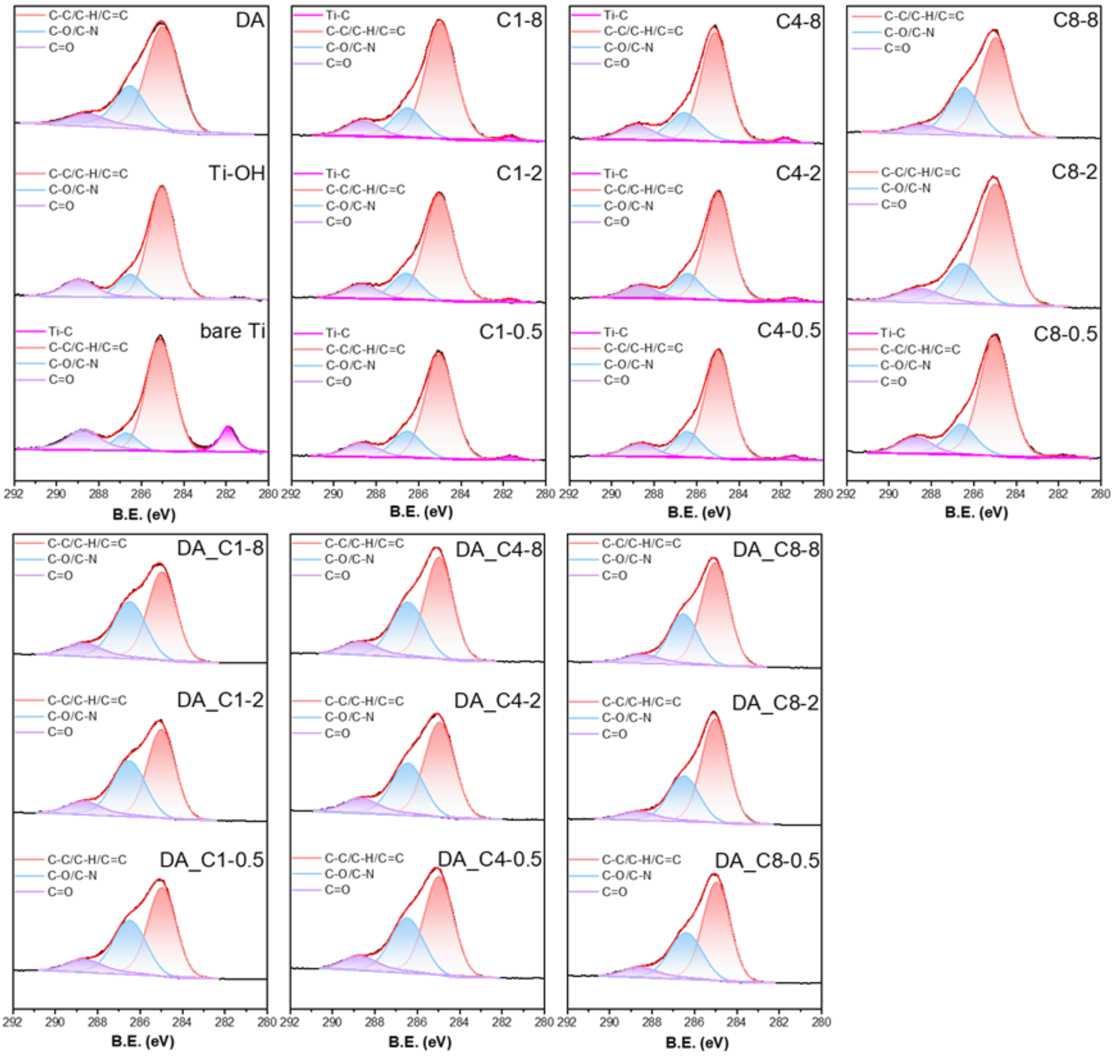

Figure S13. The C1S deconvolution spectra for different titanium substrates prepared by the single-layer and two-layer approaches.
